# Qualitative motivation with sets and relations

**DOI:** 10.3389/fpsyg.2022.993660

**Published:** 2023-01-25

**Authors:** Ali Ünlü

**Affiliations:** School of Social Sciences and Technology, Technical University of Munich, Munich, Germany

**Keywords:** self-determination theory, motivation, knowledge space theory, set, relation, inductive item tree analysis, motivational implication, dimensionality

## Abstract

In self-determination theory (SDT), multiple conceptual regulations of motivation are posited. These forms of motivation are especially qualitatively viewed by SDT researchers, and there are situations in which combinations of these regulations occur. In this article, instead of the commonly used numerical approach, this is modeled more versatilely by sets and relations. We discuss discrete mathematical models from the theory of knowledge spaces for the combinatorial conceptualization of motivation. Thereby, we constructively add insight into a dispute of opinions on the unidimensionality vs. multidimensionality of motivation in SDT literature. The motivation order derived in our example, albeit doubly branched, was approximately a chain, and we could quantify the combinatorial details of that approximation. Essentially, two combinatorial dimensions reducible to one were observed, which could be studied in other more popular scales as well. This approach allows us to define the distinct, including even equally informative, gradations of any regulation type. Thus, we may identify specific forms of motivation that may otherwise be difficult to measure or not be separable empirically. This could help to dissolve possible inconsistencies that may arise in applications of the theory in distinguishing the different regulation types. How to obtain the motivation structures in practice is demonstrated by relational data mining. The technique applied is an inductive item tree analysis, an established method of Boolean analysis of questionnaires. For a data set on learning motivation, the motivation spaces and co-occurrence relations for the gradations of the basic regulation types are extracted, thus, enumerating their potential subforms. In that empirical application, the underlying models were computed within each of the intrinsic, identified, introjected, and external regulations, in autonomous and controlled motivations, and the entire motivation domain. In future studies, the approach of this article could be employed to develop adaptive assessment and training procedures in SDT contexts and for dynamical extensions of the theory, if motivational behavior can go in time.

## 1. Introduction

Knowledge space theory (KST) was introduced by Doignon and Falmagne (1985, 1999), refer also Falmagne and Doignon (2011), which is a relatively recent psychometric theory. Initially, that theory was developed for the assessment and training of knowledge, but has evolved into a broader range of applications (e.g., Albert and Lukas, 1999; Falmagne et al., 2013). KST, as compared with the statistical item response theory (e.g., Van der Linden and Hambleton, 1997), for example, is inherently combinatorial; it did not develop from classical numerical scales in the first place (for a conceptual comparison of these theories, see Ünlü, 2007). The behavioral orientation of KST is good for qualitative modeling. Since numerical values (e.g., person ability or item difficulty) are more strongly aggregated numbers, in particular, restricted by their own natural ordering, the use of more fine-grained combinatorial structures (e.g., persons represented by their sets of skills they possess) allows for greater flexibility in more diagnostic modeling, as we will describe in this article. In a numerical approach, two persons are typically reduced to their aggregate motivation degrees, say 1.27 < 2.35, which are always comparable (real numbers). This is in contrast to representing persons by the sets of motivations they possess, which are not necessarily comparable, for example, if represented by {*a*, *c*, *e*} and {*b*, *c*, *d*}, where neither is a subset of the other. To avoid misconception at this point, obviously, there may be situations where the former perspective is desirable or sufficient for a use case, but if the aim is to have a qualitative assessment or conceptualization, the latter approach may be better suited. It depends on what the research aims are. For example, if ordering motivational behavior along the relative autonomy continuum is preferred, if continuously unidimensional, a quantitative single-valued scoring rule can suffice. However, if the goal is to see which motivations more strongly interrelate to what substantive outcomes, a qualitative model can be more useful. This article assumes that the latter perspective is desired for the problem of interest.

We describe an application of KST to self-determination theory (SDT). SDT was proposed by Deci and Ryan (1985, 2000), refer also Ryan and Deci (2000, 2002). The need for qualitative treatment of motivation was raised by researchers in SDT, in particular by Chemolli and Gagné (2014), with further pertinent SDT references therein, describing empirical problems that required a qualitative conceptualization of motivation (e.g., Koestner et al., 1996; Vansteenkiste et al., 2009). KST can contribute to this endeavor, as this article aims at demonstrating sets and relations among motivations (for advantages and a limitation, refer to section “Usefulness of this approach for motivation research and limitation”).

Why do we treat the dichotomous data case first with this article? We are aware that SDT instruments use Likert scales and that dichotomization of polytomous or continuous data or variables can be controversial, but we have the following reasons. The theory of knowledge spaces is far more advanced in the dichotomous formulation, with a plethora of easier-to-grasp and better-accessible results that can readily be applied in motivation research. For example, if you take Birkhoff’s theorem, an important mathematical as well as methodological result, this theorem is way easier to formulate and understand than its polytomous counterparts. In KST, the polytomous case is an ongoing research, still, many powerful dichotomous concepts of KST have to be generalized and developed for polytomous items, if possible. In addition, dichotomous indicators can provide useful information about binary classification problems, for example, whether a person is intrinsically motivated or not, or more generally, which motivations may or may not be present in the total motivational profile of a person. That is, dichotomous indicators can still be informative enough for such use cases involving binary decisions. Dichotomous results may also be viewed as necessary conditions for a polytomous model, for example, when violated, they may give evidence against the model. In general, results obtained by dichotomous analyses are, albeit rougher, easier to interpret. In particular, what we will see in this article is that the approach based on sets and relations in dichotomous formulation allows us to quantify, combinatorially and qualitatively, how far multidimensionality may be away from unidimensionality, thus contributing to the debated issue of dimensionality in SDT (Chemolli and Gagné, 2014; Sheldon et al., 2017). Anyhow, we have to see the merits of this approach after SDT researchers have given this method a try by testing it across their motivation scales or empirical studies.

How to derive KST relations among motivations from empirical data? We discuss one possibility based on the data-analytic approach of the inductive item tree analysis (IITA). There are other methods as well (e.g., Albert and Lukas, 1999), for example, by querying experts; theory-driven, based on skills or competencies; or by data mining (which is nearest to what we present). Publications to learn more about IITA are Schrepp (1999a,b, 2003, 2006), Sargin and Ünlü (2009), Ünlü and Sargin (2010), and Ünlü and Schrepp (2021). As a well-established method of Boolean analysis of questionnaires, IITA takes as input a data set (e.g., of motivation scores), and in this article, it is treated for the dichotomous case and derives the set of implications deemed to be plausible for the data set according to some faithful criteria. That is, detailed later, the IITA algorithm can be used to extract motivation co-occurrence relations, and thus, by application of Birkhoff’s theorem, quasi-ordinal motivation spaces, from data motivation variables.

In addition to data analysis, rather theoretically, we believe that the application of KST to SDT can in particular be useful for the representation of the logical structure of motivations. Based on mathematical considerations, we may obtain principled definitions of such pertinent concepts as self-determination and derive from these more abstract definitions, results about their universal (mathematical or axiomatic) properties in population instead of sample quantities (Ünlü, 2022).

This article has the following structure. In section “Self-determination theory,” the theory of self-determination is reviewed, and in section “Knowledge space theory, Birkhoff’s theorem, and inductive item tree analysis,” the knowledge space theory. In particular, section “Knowledge space theory, Birkhoff’s theorem, and inductive item tree analysis” also includes a short introduction to IITA and Birkhoff’s theorem. In section “Sets and relations among motivations,” the application of KST sets and relations to motivation is described. The SDT analogs of the basic concepts of KST are the motivation domain, motivation structure, motivation state, motivation co-occurrence relation, and quasi-ordinal motivation space. In section “An empirical application,” an empirical example is provided, which concerns learning motivation. In section “Usefulness of this approach for motivation research and limitation,” we outline why this approach to the modeling and analysis of motivation is useful and a limitation of this study. This article ends with a conclusion in section “Conclusion,” and with Appendices A, B containing the scale and binary data sets used for the empirical application, respectively.

## 2. Self-determination theory

Self-determination theory maintains a comprehensive website at https://selfdeterminationtheory.org. As a theory of motivation, SDT investigates what drives people to act (Ryan(ed.), 2019; Conesa et al., 2022; Gagné et al., 2022). The basic concepts of SDT are best represented by Table 1, a table very often reported in SDT publications, and here, we present a slightly modified interpretation of it.

**TABLE 1:** Self-determination continuum also called SDT’s taxonomy of motivation (cf., Deci and Ryan, 2000).

Behavior continuous	Nonself-determined	Self-determined			
Type of motivation	Amotivation	Extrinsic motivation			Intrinsic motivation
Type of regulation	Non-regulation	External regulation	Introjected regulation	Identified regulation	Intrinsic regulation
	Lack of external and internal controls and motives Complement of motivation	Constraints of external controls Lack of internal motives	Constraints of internal controls Forced external motives	Internally identify with external value Quasi-unforced internal motives	Lack of external and internal controls Unforced internal motives
Locus of causality continuous	Impersonal	External	Somewhat external	Somewhat internal	Internal
Trichotomy of form	Uncontrolled Non-autonomous	Controlled Non-autonomous	Controlled Non-autonomous	Quasi-uncontrolled Quasi-autonomous	Uncontrolled Autonomous

We see three main types of motivation (intrinsic motivation, extrinsic motivation, amotivation) with their corresponding regulatory styles (intrinsic regulation, identified regulation, introjected regulation, external regulation, non-regulation) and loci of causality (internal, somewhat internal, somewhat external, external, impersonal). These regulations describe increasingly less self-determined (with far left nonself-determined) behaviors, of different qualitative forms (uncontrolled/autonomous, quasi-uncontrolled/quasi-autonomous, controlled/non-autonomous, controlled/non-autonomous, uncontrolled/non-autonomous).

Briefly, intrinsic motivation represents behavior enjoyed doing it for its own sake, extrinsic motivation is instrumental behavior, and amotivation is the lack of motivation. SDT does not further differentiate intrinsic motivation and amotivation, thus their single regulations are interpreted accordingly. But extrinsic motivation, which is assumed, can have varying internalization. Extrinsic motivation is differentiated into three (or four) gradually internalized regulation types, in increasing order of internalities, namely, external regulation, introjected regulation, identified regulation, and integrated regulation. Again briefly, external regulation is behavior dictated by purely external factors such as reward or punishment. Introjected regulation is more internalized than external regulation and includes proving oneself worthy or avoiding guilt. Identified regulation is even more internalized than introjected regulation and is described as acting to express rational values without being accompanied by unforced interval motives such as fun or inherent satisfaction. Integrated regulation, although extrinsic motivation, is assumed to be only internal motivation (different from intrinsic regulation), located on the internalization continuum of SDT between identified regulation and intrinsic regulation. With integrated regulation, a person’s identified values are even further internalized and integrated with each other. Integrated regulation seems to be difficult to measure (e.g., Roth et al., 2009; Gagné et al., 2014). In literature, additional forms of motivation have also been proposed, for example, negative introjection (left) and positive introjection (right) in Sheldon et al. (2017) or avoidance introjection (left) and approach introjection (right) in Assor et al. (2009). In each of these cases, both forms of introjection are between external regulation and identified regulation. Probably other forms of motivation may be possible here and there. Why do we list them? In the approach based on sets and relations, which provides a general framework, all of these motivations can be easily included in the formulation of the models. These motivations define what will be called the motivation domain, the set of all motivations of interest. The set and relation representations defined are built upon the specified domain of motivations.

In this article, we show how KST can be applied to qualitatively model these motivations or regulations of SDT’s (extended) taxonomy. Instead of, typically by confirmatory factor analysis, introducing one or more latent continuous dimensions and factors, to represent each of the regulation types, we use sets and relations among those motivations or regulations. A strength of the latter approach is that it allows for very general combinatorial structures and, thus, offers more flexibility in modeling motivation qualitatively (section “Usefulness of this approach for motivation research and limitation”).

## 3. Knowledge space theory, Birkhoff’s theorem, and inductive item tree analysis

We give a short introduction to KST (Doignon and Falmagne, 1985), including the theorem by Birkhoff (1937), and IITA (Schrepp, 1999b; Sargin and Ünlü, 2009). These three components are the building blocks of the methodology used in this article. The core contributions of our study are to apply KST models to represent motivation in SDT, with equivalent mathematical representations at the levels of persons and motivations by Birkhoff’s theorem as a byproduct, and the concrete implementation of those models in data through the use of IITA.

### 3.1. Knowledge space theory and Birkhoff’s theorem

An application with examples of the concepts in motivation presented here can be found in section “Sets and relations among motivations.”

#### 3.1.1. Surmise relation

The starting point for a theory of knowledge assessment is to assume that in a knowledge domain of interest, the pieces of knowledge may imply each other. For example, in the knowledge domain of natural numbers, the mastery of the arithmetic problem *b* “3 ⋅ 2 = ” may imply the mastery of the arithmetic problem *a* “2 + 2 = ”. That is, the mastery of problem *a* is assumed to be a prerequisite for the mastery of problem *b*. Mathematically, this is represented by the ordered pair *a* ⊑ *b* of a binary relation ⊑ on the knowledge domain. In accordance with the interpretation of mastery, that relation is assumed to be reflexive and transitive, a quasi-order, or as it is also called in KST, a surmise relation.

Definition 1. Let *Q* be a non-empty and finite set of dichotomous items, the (knowledge) domain. Let ⊑ be a binary relation on *Q*, that is, a subset of *Q* × *Q*. We call ⊑ a quasi-order or surmise relation (on *Q*) if and only if it is reflexive and transitive, that is, if and only if, respectively, *x* ⊑ *x* for all *x* ∈ *Q*, and *x* ⊑ *y*, *y* ⊑ *z* implies *x* ⊑ *z* for all *x*, *y*, *z* ∈ *Q*.

Typically, *Q* can be a psychological or educational test consisting of dichotomous questions or problems that can either be solved (coded *1*) or failed (coded *0*) by examinees, and a surmise relation on that test then captures the mastery dependencies among the test items.

#### 3.1.2. Knowledge structure and space

The implications of the surmise relation entail that only certain mastery patterns, represented by subsets of the domain, are admissible, which are called the knowledge states. For example, the subset of items is mastered by a student’s, her or his, knowledge state. If it contains the multiplication item *b*, then it must also contain the addition item *a*, since *a* ⊑ *b*. The collection of all so compatible knowledge states is called the knowledge structure. In ideal conditions, if no response errors occur, the only response patterns possible would be the knowledge states.

Definition 2. Let *Q* be a domain. A knowledge structure ℋ on *Q* is a set of subsets of *Q*, which contains at least the empty set ∅ and *Q* itself. The elements of ℋ are called knowledge states.

Knowledge states are subsets of *Q*. Thus, we can take their union ∪ and intersection ∩, which yield the important special case of a knowledge structure, a quasi-ordinal knowledge space.

Definition 3. Let ℋ be a knowledge structure on the domain *Q*. We call ℋ a knowledge space (on *Q*) if and only if *G* ∪ *H* ∈ ℋ for all *G*, *H* ∈ ℋ. The knowledge structure ℋ is a closure space (on *Q*) if and only if *G* ∩ *H* ∈ ℋ for all *G*, *H* ∈ ℋ. A quasi-ordinal knowledge space is a knowledge structure that is both a knowledge space and a closure space.

#### 3.1.3. Birkhoff’s theorem

The quasi-ordinal knowledge space model is the set representation at the level of persons and the surmise relation model is the order representation at the level of items. They correspond to a person’s ability and item difficulty of numerical item response theory. In contrast to the numerical approach, in KST, the two representations are connected by a central mathematical theorem, which is Birkhoff’s theorem. The details of this theorem can be found in Falmagne and Doignon (2011, section 3.8, pp. 56–58).

Theorem 1 (Birkhoff, 1937). There exists a one-to-one correspondence between the collection of all quasi-ordinal knowledge spaces ℋ on a domain *Q* and the collection of all surmise relations ⊑ on *Q*, defined by the two equivalences, for *p*, *q* ∈ *Q*, *H* ⊆ *Q*,


p⊑q :⟺(∀G∈ℋ,q∈G⟹p∈G),

H∈ℋ :⟺(∀r⊑s,s∈H⟹r∈H).

This theorem mathematically links two different levels of empirical interpretations. It will be applied to SDT and exemplified in section “Sets and relations among motivations.”

#### 3.1.4. Validation

Validation procedures were proposed in the literature based on probabilistic extensions of knowledge structures. The most prominent one is the basic local independence model, a latent class scaling model (Doignon and Falmagne, 1999, chapter 7). For logistic and generalized normal ogive statistical validation procedures in KST, refer to Stefanutti (2006) and Ünlü (2006), respectively. Latent class analysis exploratory, estimation, and testing procedures were also studied for knowledge structures (Schrepp, 2005; Ünlü, 2011). For recent developments in performance- and competence-based knowledge space theory, including further, also more qualitative, validation procedures, refer to Falmagne et al. (2013).

### 3.2. Inductive item tree analysis

In IITA, competing relations are generated and a fit measure is computed for each of these relations in order to find that relation which most adequately describes the data. Since traditional inference-based methods, such as (asymptotic) chi-squared goodness-of-fit tests (available as well), do almost always reject the wished model (placed in the null hypothesis), this class of IITA relational mining techniques is generally effective and more useful. The R (The R Core Team, 2022) package DAKS (Ünlü and Sargin, 2010) freely available at https://CRAN.R-project.org/package=DAKS implements the IITA procedures, in addition to software by Schrepp (2006).

#### 3.2.1. General problem

What is the general problem addressed by IITA? Assume that you have noisy indicators for latent variables of interest and that among those latent variables there exist latent logical, that is, deterministic, implications. The goal of IITA is to detect these implications from the information on the indicators. A typical KST example of an implication, refer section “Knowledge space theory and Birkhoff’s theorem,” is that the mastery of a math problem may imply the mastery of another math problem, where the questions of a math test are the indicators. In section “Sets and relations among motivations,” we apply this idea to motivation in SDT. There, the indicators are the test items of a motivation questionnaire (https://selfdeterminationtheory.org/questionnaires), which measure their underlying, for example, intrinsic and external regulations. Then, a logical implication between motivations assumes that the latent possession or occurrence of a motivation implies the possession or occurrence of another motivation. If in a study such motivational implications are deemed to be realistic or of interest, IITA is a technique that can be used to uncover those implications from the motivation questionnaire data.

#### 3.2.2. Computational components

The IITA algorithm consists of three computational components. First, it constructs a selection set of competing quasi-orders on the domain of latent (e.g., motivation) variables. That construction is inductive. For varying numbers of observed counterexamples of an (e.g., motivational) implication (premises true but conclusions false), anchored with the simplest quasi-order consisting of all implications that are not violated in the data, in successive steps, quasi-orders (e.g., motivation co-occurrence relations) are constructed. This is realized by adding specific implications that have no more than the predefined numbers of counterexamples in the data set and that do not violate transitivity. In this way, a maximum of sample size plus one, typically a much smaller number than this, of increasingly more complex quasi-orders are derived. Second, the fit of each constructed quasi-order to the data set is quantified by a measure of the average squared differences between the observed and under the model expected numbers of counterexamples for the implications. Third, a best-fitting (e.g., motivation co-occurrence) relation of the selection set with the computed minimum discrepancy is chosen as the final solution. Subsequently, we outline these components of the technique, and more details can be found in Ünlü and Schrepp (2021, section 2).

#### 3.2.3. Inductive construction

We start with notation. Let *Q* = {*i*_1_, …, *i*_*m*_} be the domain of *m* ≥ 2 dichotomous items. Let *D* = {*d*_1_, …, *d*_*N*_} be the data set (with repetitions) of observed response patterns (mappings) *d*_*n*_ : Q → {0, 1}, where *d*_*n*_ (*i*) = *0* or 1 stands for the response of the subject *n* = 1, …, *N* to the item *i* ∈ *Q*. For *i*, *j* ∈ *Q*, let *b*_*ij*_ = |{*d* ∈ *D* : *d*(*i*) = 1 ∧ *d*(*j*) = 0}| be the number of subjects of the sample who solved item *i* but failed item *j*. If we postulate the implication *i* ⟶ *j* (Definition 5), *b*_*ij*_ is the number of observed counterexamples for that implication. We can define the binary relation ⊑_0_ of all implications that are not violated in data *D* by *j*⊑_0_*i* :⟺ *b*_*ij*_ = 0 for all *i*, *j* ∈ *Q*. This relation ⊑_0_ is a quasi-order on *Q* (Van Leeuwe, 1974). However, this is not the final quasi-order that IITA returns. To accept ⊑_0_ is generally not satisfactory since this is data fitting, which does not account for response errors. Thus, IITA allows for varying numbers of observed counterexamples *L* = 0, 1, …, *N*.

The construction of the quasi-orders is as follows. The IITA algorithm starts with ⊑_0_, but inductively constructs bigger quasi-orders. The procedure to construct the *L* + 1 step quasi-order ⊑_*L*+1_ from the *L* step quasi-order ⊑_*L*_, anchoring with ⊑_0_, for *L* = 0, 1, …, *N* − 1, consists of the following steps 1, 2, and 3:

1. To determine the set *A*_*L+1*_ of all item pairs that are not already contained in ⊑_*L*_ and have no more than *L + 1* observed counterexamples in *D*.
2. To iteratively repeat the following two operations a and b:
  a. For each element of *A*_*L+1*_, check if it violates transitivity in ⊑_*L*_ ∪ *A*_*L*+1_. If so, mark that element.
  b. If no element is marked in operation a, stop step 2. Otherwise, delete all marked elements from *A*_*L+1*_ and restart the process in operation a with this reduced new set *A*_*L+1*_.
3. When the process in step 2 stops, the remaining implications in *A*_*L+1*_ do not violate transitivity in ⊑_*L*_ ∪ *A*_*L*+1_. By construction, ⊑_*L*+1_ := ⊑_*L*_ ∪ *A*_*L*+1_ is the *L* + 1 step quasi-order.

Thus, by IITA, the increasingly bigger quasi-orders ⊑_0_ ⊆ ⊑_1_ ⊆ … ⊆ ⊑_*N*_ are inductively constructed.

#### 3.2.4. Fit measure

Among these relations, IITA proposes the following method to determine the best-fitting quasi-order. We quantify the fit of any of the IITA quasi-orders ⊑_*L*_, *L* = 0, 1, …, *N* to the data set *D* by the measure:


$$\text{diff}({⊑}_{L},D)=\frac{\sum_{i\neq j}{\left(b_{ij}-t_{ij}\right)}^{2}}{m\left(m-1\right)},$$

where the sum is taken over all item pairs (*j*, *i*) ∈ *Q* × *Q*, *i* ≠ *j*, and *m* is the number of items. In addition, *b*_*ij*_ is the observed number of subjects who solved item *i* but failed item *j*, and *t*_*ij*_ is the corresponding theoretical value expected and derived under the assumption that ⊑_*L*_ is the correct quasi-order underlying the data set *D*.

The derivation of the *t*_*ij*_ estimators is intricate and necessitate a few considerations.

1. Assume that ⊑_*L*_, for a given *L*, is the quasi-order of true logical implications between the items. How many violations for a true implication *i*⟶*_L_j*, *i*, *j* ∈ *Q* can we expect? If we assume a single response error probability by which a true implication may be violated, that rate can be estimated by:


$$\gamma_{L}=\frac{\sum_{𝒢}\frac{b_{ij}}{p_{i}N}}{\left|𝒢\right|}\text{if}𝒢\neq \emptyset ,\text{or}0\text{if}𝒢=\emptyset ,$$

where *𝒢* = {*j*⊑*_L_i* : *i* ≠ *j* ∧ *p*_*i*_ ≠ 0}, and $p_{i}=\frac{\left|\left\{d\in D:d\left(i\right)=1\right\}\right|}{N}$, *i* ∈ *Q* is the relative frequency of subjects of the sample who solved item *i*. Thus, γ_*L*_ is an estimated average amount of random response errors in the data, under the assumption that ⊑_*L*_ is the underlying true quasi-order. For further motivation for this choice of estimator, refer to Ünlü and Schrepp (2021).

2. Under the assumption that ⊑_*L*_ is the correct quasi-order, thus based on the corresponding estimated error probability γ_*L*_, for any item pair (*j*, *i*) ∈ *Q* × *Q*, *i≠j*, the, under ⊑_*L*_ derived, theoretical values *t*_*ij*_ used in the definition of the diff measure can be estimated as follows: three cases are distinguished (for more details, refer to Ünlü and Schrepp, 2021). First, if *j*⊑*_L_i*, *i≠j*, use the estimation equation *t_ij_* = γ*_L_p_i_N*. Second, if *j*⋢*_L_i* and *i*⋢*_L_j*, assume that the items are stochastically independent, and set *t*_*ij*_ = (1 − *p*_*j*_)*p*_*i*_*N*. Third, if *j*⋢*_L_i* but *i*⊑*_L_j*, the estimator is *t*_*ij*_ = *max*(0, (*p*_*i*_ − *p*_*j*_ + *p*_*j*_γ_*L*_)*N*).

#### 3.2.5. Selection

A validation procedure is obtained that gives information about which model to pick. With the above ingredients, in data *D*, the fit measure diff is computed for each quasi-order obtained by inductive construction. Thus, a non-negative real value is associated with any of the competing quasi-orders. Since diff quantifies an average squared difference between observed and expected variables, smaller values of the measure are interpreted to indicate a better fit. In particular, the decision rule is to select that quasi-order among ⊑_0_, ⊑_1_, …, ⊑_*N*_, which has the minimum diff value. This is the final solution returned by the IITA algorithm.

## 4. Sets and relations among motivations

We apply the basic concepts of KST to SDT and describe them in motivation.

Before presenting the definitions, a general remark is in order. The program of this article has far-reaching consequences (section “Usefulness of this approach for motivation research and limitation”). The KST models applied to motivation can be used to develop routines in motivation research for the adaptive assessment and training of motivation using computers. Adaptive testing is the major strength and point of origin of KST (Falmagne et al., 2013, with references therein). Noteworthy, there is an essential difference between the educational vs. motivational applications of the models. The number of conceivable states of motivation in SDT may not be that large as compared with the states of knowledge studied in KST, with several million feasible knowledge states in large-scale empirical studies. This is clearly an advantage of the SDT application, in particular, for combinatorial as well as statistical reasons.

We want to motivate the basic concepts by an example, and then give the definitions for motivation. Consider the regulations, for ease of presentation with no gradations of the regulations (generalized below), external regulation *a*, introjected regulation *b*, identified regulation *c*, and intrinsic regulation *d*. These motivations make up the motivation domain of interest, that is, the set of all considered motivations *M* = {*a*, *b*, *c*, *d*} (other choices for the domain are discussed later). The central assumption is that these motivations can only occur in certain combinations in the population of reference, called the motivation states, which are subsets of the motivation domain. For example, a student could be externally and introjectedly motivated at the same time, represented by the state or subset {*a*, *b*} of the motivation domain. Or, this is an assumption that can change depending on the model used; for a student to be intrinsically motivated, the student necessarily needs to have the other three motivations. In this case, *M* = {*a*, *b*, *c*, *d*} is the only possible motivation state containing intrinsic regulation, and other combinations containing intrinsic regulation such as {*a*, *d*} (externally and intrinsically motivated) or {*d*} (only intrinsically motivated) are excluded according to the posited model. Since amotivation is understood to be the state of no motivation, this could be modeled by a specific subset, the empty set ∅. We can collect together all feasible combinations of motivations, the motivation states, into a set on its own, called the motivation structure. In this example, the combinations {*a*, *b*} and *M* belong to the motivation structure, but not {*a*, *d*} and {*d*}. As mentioned, if this model is deemed to be empirically inadequate, it can be replaced by another model; the mathematical definition allows us to flexibly define the states, that is, the combinations of motivations considered to be feasible or occurring in an empirical study. The practical derivation of the motivation structure can be (inclusive “or”) theory-driven, derived from querying experts, or obtained by statistics and data analysis, where the latter is the approach pursued with this article. Let us assume that a researcher has identified the following motivation structure in the motivation domain of her study,


(1) ℳ={∅,{a},{b},{a,b},{b,c},{a,b,c},M}.

This motivation structure captures the logical organization of the motivations of interest in the population of reference. Only those combinations of the motivations are feasible, in the sense that they have shares of the population. Mathematically though, the motivation structure is defined to always include the empty set and the motivation domain, for technical reasons. Leaving out these extreme states from the definition of a motivation structure, in principle you could, will complicate, or probably invalidate, mathematical results, at least in their common formulations. We will stay with the definition used in KST and include as stated always the empty set and the motivation domain itself. So, amotivation, if modeled as the empty set, in contrast to the element(s) of the domain, and the possibility of possessing all regulations jointly are assumed to be motivation states under any deterministic model. Thus, if these extreme motivation states do not occur, any specified deterministic model will be wrong in those states. Otherwise, the deterministic model can always be chosen to be correct in all other states. However, this “methodological artifact” is not restrictive from an empirical viewpoint. In practice, if any of these two states is deemed to be empirically implausible, its probability of occurrence in the population of reference will (virtually) be zero, thus it will be discarded in the probabilistic formulation and extension of the deterministic model. However, as seen below, at the level of motivations viewed as “items” composing the states, this subtle issue is resolved and does not appear.

**FIGURE 1:**
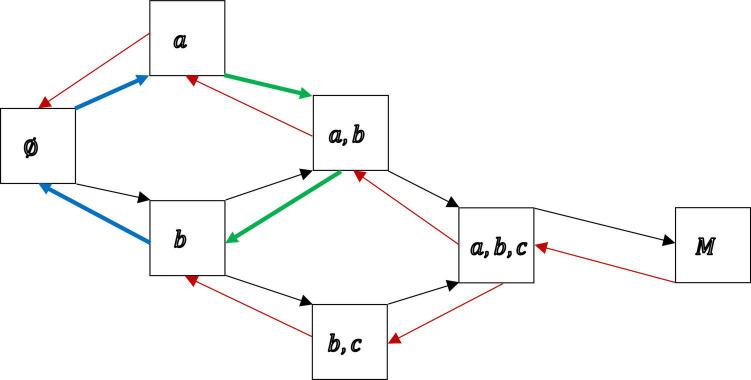
The directed graphs, in black and red, respectively, of the motivation structure ℳ = {∅, {*a*}, {*b*}, {*a*, *b*}, {*b*, *c*}, {*a*, *b*, *c*}, *M*}, with *M* = {*a*, *b*, *c*, *d*}. A black arc (directed edge) *A* ⟶ *B* linking the motivation state *A* (left) to the motivation state *B* (right) means *A* ⊂ *B* (*A* subset of *B*) and there is no other motivation state between the two. A red arc *B* ⟶ *A* linking the motivation state B (right) to the motivation state A (left) stands for *B* ⊃ *A* (*B* superset of *A*) and there is no other state in between. A trajectory describing the transition from the state of pure external motivation {*a*} to the state of pure introjected motivation {*b*}, along directed edges (of both colors) in only states of the motivation structure, is shown in green, {*a*} ⟶ {*a*, *b*} (black arc) and {*a*, *b*} → {*b*} (red arc). Another trajectory, now from state {*b*} to state {*a*}, posited under this model, probably critical empirically, is to become amotivated first, purging the existent introjected motivation, before the other external motivation can be gained, shown in blue. In particular, there is no direct arc linking the two motivation states, thus, according to this model, external motivation cannot be directly converted into introjected motivation and vice versa, but instead, for example, under the green trajectory, must be attained jointly first before the initial one is forfeited to end up with the other. In these graphs, motivations are gained or lost one by one in progressions from state to state. Restrictions of this sort imply serviceable mathematical properties.

The motivation structure ℳ in Eq. 1 induces the two directed graphs shown in Figure 1, which suggests a process of how motivation may progress from state to state, for example, over time. Figure 1 helps to indicate the general idea, and in particular, the flexibility that comes with such a discrete mathematical structure. Depending on the empirical situation, a proper model could be specified (e.g., quantitatively by exploratory data analysis, below), allowing for the qualitative, or even dynamic over time, analysis of motivation.

With motivation progression, things can get mathematically more tractable, if only “learning” is possible. That is, if only additional, new motivations are attained, one by one, and no motivations are lost during progression, moving from left to right in the graph of Figure 1, along the black arcs only. In such a more restrictive model, in this example, a student cannot reach the states of pure external motivation or pure introjected motivation if she or he is initially only introjectedly or externally motivated, respectively. This would be prohibited by model assumption. This case of only “learning,” that is, merely moving from left to right along black arcs, cumulative in the “items” or motivations “learned,” one at a time, is the case that has been extensively studied in dichotomous KST. It is especially interesting, if justifiable in a study on motivation, since it entails a rich mathematical theory, implying strong mathematical measurement properties (Falmagne and Doignon, 2011).

Here is the definition in terms of motivation.

Definition 4. Let *M* be a non-empty set of motivations or regulations (examples below). Let ℳ be a collection of subsets of *M*, which contains at least ∅ and *M*. Then, ℳ is called a motivation structure on (or in) the motivation domain *M*. The elements of ℳ are the motivation states.

Example 1. We consider the general case. The motivation domain *M* could consist of *k* forms (gradations) of non-regulation *a*_1_, …, *a*_*k*_; *l* forms of external regulation *e*_1_, …, *e*_*l*_; *m* forms of introjected regulation *j*_1_, …, *j*_*m*_; *n* forms of identified regulation *d*_1_, …, *d*_*n*_; *u* forms of integrated regulation *g*_1_, …, *g*_*u*_; and *o* forms of intrinsic regulation *i*_1_, …, *i*_*o*_. In this case, the extreme motivation state *M* describes hypothetical behavior that is regulated by all types jointly. The other extreme state ∅ may represent a sort of totally unregulated behavior, unexplainable by any of the types (regarding the extreme states, refer to the aforementioned text). The motivation structure in this domain can contain arbitrary combinations of these regulations, such as the state consisting of the last form of external regulation and the first two (if *o* ≥ 2) of intrinsic regulation {*e*_*l*_, *i*_1_, *i*_2_}, which represents a student extrinsically and intrinsically motivated in respective gradations of the regulations.

Example 2. Let the notation be as in Example 1. Assume that the motivations are not further graded, *k* = *l* = *m* = *n* = *o* = 1, where integrated regulation is not of interest (in the sequel). If non-regulation *a*, external regulation *e*, introjected regulation *j*, identified regulation *d*, and intrinsic regulation *i* can only occur separately,


$${ℳ}_{1}=\left\{\emptyset ,\left\{a\right\},\left\{e\right\},\left\{j\right\},\left\{d\right\},\left\{i\right\},M\right\}.$$

If, in this representation, the intermediate introjected and identified regulations have cumulative gradations *j*_1_≼_*j*_…≼*_j_jm* and *d*_1_≼_*d*_…≼_d_*d*_*n*_, indexed in the increasing rankings of their linear orderings ≼_*j*_ and ≼_*d*_ (orders are treated later),


$$\begin{array}{l} {ℳ}_{2}=\{\emptyset ,\{\text{a}\},\{\text{e}\},\{j_{1}\},\{j_{1},j_{2}\},\ldots ,\{j_{1},j_{2},\ldots ,j_{m}\},\\ \;\{d_{1}\},\{d_{1},d_{2}\},\ldots ,\{d_{1},d_{2},\ldots ,d_{n}\},\{\text{i}\},\text{M}\}. \end{array}$$

**FIGURE 2:**
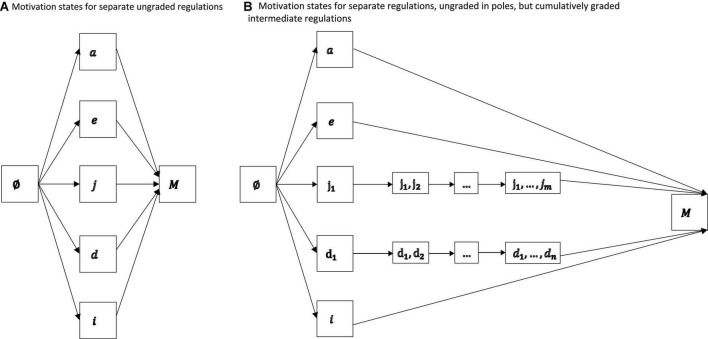
Motivation states for separate ungraded regulations **(A)** and separate but cumulatively graded intermediate regulations **(B)**. In both representations, non-regulation, external regulation, and intrinsic regulation are singletons. The linear orders imposed on the gradations of introjected regulation and identified regulation could be associated with the varying cumulative internalities that the gradations may have along an internalization continuum.

We present the corresponding directed graphs of Example 2 in Figure 2.

Besides motivation structures, the set representation, there is the other representation based on relations. We can adumbrate how the two representations are connected by reconsidering the example with ℳ = {∅, {*a*}, {*b*}, {*a*, *b*}, {*b*, *c*}, {*a*, *b*, *c*}, *M*} in Eq. 1. These states are the combinations of motivations that people can have. Under this model, if a person possesses motivation *c*, that is, if she or he is in one of the states {*b*, *c*}, {*a*, *b*, *c*}, or {*a*, *b*, *c*, *d*}, then this person must also possess motivation *b*, since *b* is in all motivation states that contain *c*. That is, (for any person) if motivation *c* occurs/is possessed, then motivation *b* occurs/is possessed. We also express this by saying that motivation *c* implies motivation *b* (always in the interpretation of occurrence or possession), denoted by *c* ⟶ *b* or *b* ⊑ *c*. Similarly, we see that if motivation *d* is possessed, then necessarily all other motivations must also be possessed, that is, *d* implies *a*, *b*, and *c*; for, the only state containing *d* is *M*, which contains all other motivations. Thus, *a*, *b*, *c* ⊑ *d*. In addition, the motivations *a* and *b* do not imply each other, since the motivation states {*a*} and {*b*} contain the motivations *a* and *b*, but not *b* and *a*, respectively. Note that this does not exclude that both motivations can occur jointly, for example, with state {*a*, *b*}. In this way, we inspect all pairs of motivations to determine whether these motivations are in relation or not, thereby yielding the following motivational implications or relation pairs *a* ⊑ *d* and *b* ⊑ *c* ⊑ *d* (including *b* ⊑ *d*). Thus, this construction, which is one part of Birkhoff’s theorem, the direction from set to relation, is concrete. The other direction from relation to set is also comprehensible and accordingly obtained. We define those subsets of the motivation domain to be motivation states that are consistent with all implications or pairs of the relation. For example, given the above relation ⊑, since *d* implies *a*, *b*, and *c*, we cannot take {*a*, *d*} to be a motivation state since it is not consistent with the relation ⊑, which requires that if *d* is possessed, then all of the other motivations must also be possessed. This subset {*a*, *d*} of the motivation domain contains *d*, but not the required, implied, motivations *b* and *c*. Since motivation *a* has no prerequisite motivation in the relation ⊑, that is, since it does not imply any other motivation, it can occur as the only motivation. That is, {*a*} is consistent with ⊑, and thus, a derived motivation state. In this way, we can check for any subset of the motivation domain whether this subset contains with any of its motivations also all of the motivations implied by this motivation in the underlying relation. This constitutes the other part of Birkhoff’s theorem. In accordance with the interpretation of motivation in possession or occurrence, we call this relation ⊑ corresponding to ℳ, which is the collection of all these derived pairs of motivations, a motivation co-occurrence relation (formally defined below).

Basically, the two representations are mathematically equivalent, but empirically they are interpreted at two different levels. From a practical viewpoint, the representation based on motivation structures is at the level of persons, whereas the co-occurrence relation is at the level of motivations “viewed as items.” What do we mean by this? A motivation structure describes the feasible combinations of the motivations people can have. An element of the structure is the motivation state of a person, a collection of regulations that jointly characterize a person. In contrast, the representation based on relations asks for valid hierarchical dependencies, a relation ⊑, among the regulations, similar to ordering items, for example, by item difficulty. For a pair of motivations *x* and *y*, we set *x* ⊑ *y*, if possessing motivation *y* entails possessing motivation *x*. This could result from or include, for example, when temporally one motivation (*x*) is attained before or at the same time as the other motivation (*y*). This implicational interpretation of motivation is general. In fact, if the concept of motivation structure is deemed to be empirically plausible, this entails the plausibility of implications between the motivations as the two formulations are connected mathematically as well as by interpretation. In addition, a special case of co-occurrence relation is the trivial relation, the diagonal, according to which no implications between the regulations are assumed, except for the reflexive implications, which are tautologies. Thus, mathematically also this case of completely unrelated regulations is contained in the definition of motivation co-occurrence relation.

Definition 5. Let *M* be the motivation domain. Assume that we can form pairs (*x*, *y*) of the regulations of the domain (e.g., if possession of *y* implies the possession of *x*). Let the set of all these pairs of regulations be denoted as ⊑. For a pair (*x*, *y*) in ⊑, we can also write *x* ⊑ *y*. We call this set ⊑ a motivation co-occurrence relation if and only if it has the following additional properties, for any choice of regulations *x*, *y*, *z* of the domain:

1. *x* ⊑ *x* (reflexivity);
2. if *x* ⊑ *y* and *y* ⊑ *z*, then *x* ⊑ *z* (transitivity).

That is, a motivation co-occurrence relation is a quasi-order (e.g., Davey and Priestley, 2002) on the motivation domain. If *x* ⊑ *y*, we say that *y* implies *x*, and write y ⟶ *x*.

The axioms of reflexivity and transitivity are empirically necessary. It is obvious that under the motivation possession interpretation, the properties of reflexivity and transitivity necessarily hold. Reflexivity is logically trivial. Possession of *x* implies the possession of *x*. We must also have transitivity. If possession of *z* implies the possession of *y*, and possession of *y*, in turn, implies the possession of *x*, then possession of *z* must also imply the possession of *x*. Thus, if we derive implications between motivations consistently in this interpretation, the resulting relationship will be reflexive and transitive, a motivation co-occurrence relation.

The two representations based on motivation structure and motivation co-occurrence relation are basically equivalent. You have to additionally assume that the motivation structure is closed under (set) union and intersection.

Definition 6. Let ℳ be a motivation structure. A motivation structure is closed under union or intersection if the unions or intersections of motivation states are again motivation states, respectively. We call the motivation structure ℳ a motivation space if ℳ is closed under union. The motivation structure ℳ is called a motivation closure space if ℳ is closed under intersection. If the motivation structure ℳ is closed under union and intersection, ℳ is called a quasi-ordinal motivation space. That is, quasi-ordinal motivation spaces are motivation spaces and motivation closure spaces.

In this article, because of Birkhoff’s theorem, we are mainly concerned with quasi-ordinal motivation spaces.

Example 3. The motivation structures ℳ_1_ and ℳ_2_ in Example 2 are motivation closure spaces, but not motivation spaces, and thus, not quasi-ordinal motivation spaces. The motivation structure ℳ in Eq. 1 is a quasi-ordinal motivation space.

**FIGURE 3:**
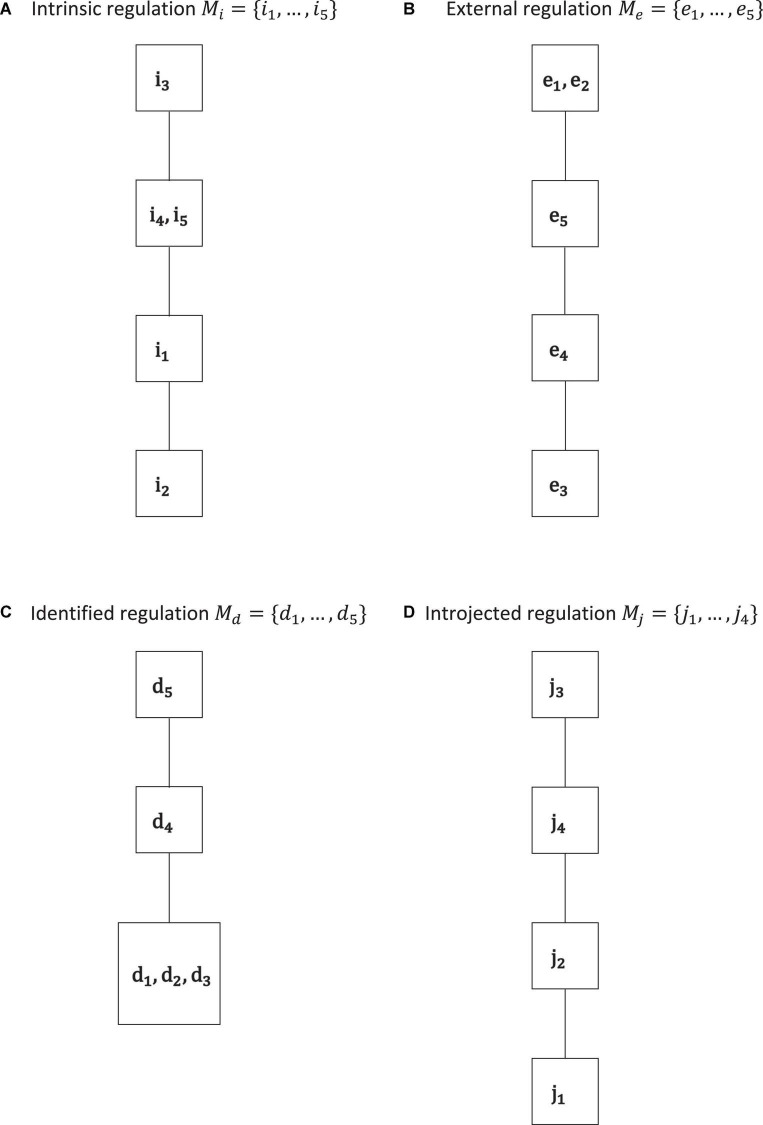
Motivation co-occurrence relations obtained by IITA of the empirical data set for the types of intrinsic regulation **(A)**, external regulation **(B)**, identified regulation **(C)**, and introjected regulation **(D)**. In each type, we see gradations that are cumulative, in the sense that they form linear orders or chains. Except for introjected regulation, there are equally informative gradations.

We recap the old, but important theorem by Birkhoff (1937) informally, which allows us to switch between the two representations of motivation, on the one hand as a motivation structure and on the other as a motivation co-occurrence relation. In applications, you could choose between the two representations depending on the focus of the study. For example, if progressions in motivation states of persons during dynamic motivation assessment are tracked (Figure 1), the representation by sets can be adequate. Or, if dependencies between motivations regarding their occurrences are of interest (Figure 3), the representation by relations is more useful. We loosely present Theorem 1 in its interpretation in motivation as a corollary, but the corresponding constructions were discussed in the example before.

Corollary 1. Let *M* be any motivation domain. For each motivation structure on *M*, which is a quasi-ordinal motivation space, you can construct a unique motivation co-occurrence relation on *M*, which is, in the above coherent sense, consonant with this space. Vice versa, for each motivation co-occurrence relation on *M*, a corresponding unique and consonant quasi-ordinal motivation space on *M* can be constructed. Since this is bijectively possible, meaning the two constructions define inverse transformations to each other, respecting the particular discrete properties, there is no loss of combinatorial information when changing from one representation to the other.

## 5. An empirical application

In this section, we discuss an illustrative example of the approach based on sets, relations, and IITA. We used data kindly provided by Müller et al. (2007). The data comprise the learning motivation subscale scores of Austrian pupils in different school class subject areas. Investigated were five items each for the intrinsic regulation and external regulation subscales, and five and four items for the intermediate identified regulation and introjected regulation subscales, respectively. For further information about the scale and for the data sets of the empirical application, see Appendices A, B. Free software to run IITA analyses can be found in Schrepp (2006) and Ünlü and Sargin (2010).

In the original data, the scores ranged from (integer) 1 − 5, including missing values, with a higher score indicating a higher level of endorsement of a regulation. The data were dichotomized relative to the center at the mid-value *3* (balanced score) of the scale. The scores *x* ≤ 2 (non-affirmative scores) were set to *0* and scores *x* ≥ 4 (affirmative scores) were set to *1*. Thus, the dichotomous score *0* encoded the negative manifest response (not endorsed regulation) and negative latent response (not possessed regulation), and *1* the positive manifest response (endorsed regulation) and positive latent response (possessed regulation). Response patterns containing at least one balanced score of *3* or missing values were discarded. The sample sizes for the used subsets of motivations ranged from *N* = 180 up to 1, 168 cases for all motivation variables altogether or only the introjected subscale, respectively (Table 2). For all resulting data sets, refer to Appendix B.

**TABLE 2:** Frequency distributions of the dichotomous scores across motivation variables of, from top to bottom, the individual regulation types, of autonomous motivation and controlled motivation, of the entire motivation domain, and the subsets *A*, *B*, and *C* of the data set, respectively.

*N* = 948	*i*_1_	*i*_2_	*i*_3_	*i*_4_	*i*_5_														
0	419	381	442	429	427														
1	529	567	506	519	521														
*N* = 1028	*e*_*1*_	*e*_*2*_	*e*_*3*_	*e*_*4*_	*e*_*5*_														
0	795	789	382	465	609														
1	233	239	646	563	419														
*N* = 1073	*d*_*1*_	*d*_*2*_	*d*_*3*_	*d*_*4*_	*d*_*5*_														
0	302	277	300	332	662														
1	771	796	773	741	411														
*N* = 1168	*j*_*1*_	*j*_*2*_	*j*_*3*_	*j*_*4*_															
0	574	675	806	779															
1	594	493	362	389															
*N* = 482	*i*_*1*_	*i*_*2*_	*i*_*3*_	*i*_*4*_	*i*_*5*_	*d*_*1*_	*d*_*2*_	*d*_*3*_	*d*_*4*_	*d*_*5*_									
0	195	171	207	196	195	136	138	150	168	285									
1	287	311	275	286	287	346	344	332	314	197									
*N* = 550	*e*_*1*_	*e*_*2*_	*e*_*3*_	*e*_*4*_	*e*_*5*_	*j*_*1*_	*j*_*2*_	*j*_*3*_	*j*_*4*_										
0	425	418	225	276	342	289	326	384	378										
1	125	132	325	274	208	261	224	166	172										
*N* = 180	*i*_*1*_	*i*_*2*_	*i*_*3*_	*i*_*4*_	*i*_*5*_	*d*_*1*_	*d*_*2*_	*d*_*3*_	*d*_*4*_	*d*_*5*_	*j*_*1*_	*j*_*2*_	*j*_*3*_	*j*_*4*_	*e*_*1*_	*e*_*2*_	*e*_*3*_	*e*_*4*_	*e*_*5*_
0	77	69	85	79	76	55	54	56	64	110	89	101	114	113	122	123	61	83	92
1	103	111	95	101	104	125	126	124	116	70	91	79	66	67	58	57	119	97	88
*N* = 342	*i*_*1*_	*i*_*3*_	*i*_*4*_	*i*_*5*_	*d*_*5*_	*j*_*1*_	*j*_*2*_	*j*_*3*_	*j*_*4*_	*e*_*3*_	*e*_*4*_	*e*_*5*_							
0	166	182	171	172	228	181	201	231	234	120	163	206							
1	176	160	171	170	114	161	141	111	108	222	179	136							
*N* = 577	*i*_*1*_	*i*_*2*_	*i*_*3*_	*i*_*4*_	*i*_*5*_	*d*_*1*_	*d*_*2*_	*d*_*3*_	*d*_*4*_										
0	224	197	239	228	227	156	159	172	191										
1	353	380	338	349	350	421	418	405	386										
*N* = 706	*d*_*5*_	*j*_*1*_	*j*_*2*_	*j*_*3*_	*j*_*4*_	*e*_*1*_	*e*_*2*_	*e*_*5*_											
0	516	403	447	514	503	567	567	465											
1	190	303	259	192	203	139	139	241											

The sample sizes *N* used in their corresponding IITA analyses of these motivation variables are also shown.

**TABLE 3:** The scale (Müller et al., 2007) used for the empirical application (Appendix A).

Ich arbeite und lerne in diesem Fach, …	Stimmt völlig	Stimmt eher	Stimmt teils/teils	Stimmt eher nicht	Stimmt überhaupt nicht
1 … weil es mir Spaß macht.	O	O	O	O	O
2 … weil ich möchte, dass mein Lehrer denkt, ich bin ein/e gute/r Schüler/in.	O	O	O	O	O
3 … um später eine bestimmte Ausbildung machen zu können (z.B. Schule, Lehre oder Studium).	O	O	O	O	O
4 … weil ich sonst von zu Hause Druck bekomme.	O	O	O	O	O
5 … weil ich neue Dinge lernen möchte.	O	O	O	O	O
6 … weil ich ein schlechtes Gewissen hätte, wenn ich wenig tun würde.	O	O	O	O	O
7 … weil ich damit mehr Möglichkeiten bei der späteren Berufswahl habe.	O	O	O	O	O
8 … weil ich sonst Ärger mit meinem/r Lehrer/in bekomme.	O	O	O	O	O
9 … weil ich es genieße, mich mit diesem Fach auseinander zu setzen.	O	O	O	O	O
10 … weil ich möchte, dass die anderen Schüler/innen von mir denken, dass ich ziemlich gut bin.	O	O	O	O	O
11 … weil ich mit dem Wissen im Fach später einen besseren Job bekommen kann.	O	O	O	O	O
12 … weil ich sonst schlechte Noten bekomme.	O	O	O	O	O
13 … weil ich gerne Aufgaben aus diesem Fach löse.	O	O	O	O	O
14 … weil ich mich vor mir selbst schämen würde, wenn ich es nicht tun würde.	O	O	O	O	O
15 … weil ich die Sachen, die ich hier lerne, später gut gebrauchen kann.	O	O	O	O	O
16 … weil ich es einfach lernen muss.	O	O	O	O	O
17 … weil ich gerne über Dinge dieses Faches nachdenke.	O	O	O	O	O

This is a copy of page 2 in https://ius.aau.at/wp-content/uploads/2016/01/mui_fragebogen.pdf (as of 30 October 2022). Since this scale was only validated in German, thus to avoid introducing any artifact, we have abstained from translating it into English.

**TABLE 4A:** Intrinsic regulation, with *N* = 948, *k* = 5 items, and *u* = 25 unique response patterns (Appendix B).

Pattern	Frequency
00000	360
00001	3
00010	4
00100	2
00101	1
00111	2
01000	34
01001	6
01010	1
01011	1
01100	1
01110	1
01111	3
10000	3
10011	1
10101	1
10111	4
11000	7
11001	3
11010	3
11011	16
11100	6
11101	2
11110	5
11111	478

**TABLE 4B:** External regulation, with *N* = 1028, *k* = 5 items, and *u* = 30 unique response patterns (Appendix B).

Pattern	Frequency
00000	233
00001	46
00010	44
00011	28
00100	100
00101	32
00110	118
00111	110
01000	2
01001	1
01010	4
01011	4
01100	7
01101	5
01110	30
01111	31
10000	5
10010	4
10011	6
10100	12
10101	7
10110	18
10111	26
11000	1
11010	1
11011	3
11100	7
11101	7
11110	23
11111	113

**TABLE 4C:** Identified regulation, with *N* = 1073, *k* = 5 items, and *u* = 29 unique response patterns (Appendix B).

Pattern	Frequency
00000	197
00001	26
00010	9
00011	8
00100	2
00101	1
00110	4
00111	2
01000	1
01001	1
01100	5
01101	1
01110	21
01111	24
10000	12
10001	4
10010	4
10011	1
10100	2
10101	2
10111	3
11000	21
11001	6
11010	8
11011	2
11100	37
11101	14
11110	339
11111	316

**TABLE 4D:** Introjected regulation, with *N* = 1168, *k* = 4 items, and *u* = 16 unique response patterns (Appendix B).

Pattern	Frequency
0000	458
0001	21
0010	7
0011	3
0100	41
0101	33
0110	3
0111	8
1000	122
1001	14
1010	32
1011	18
1100	63
1101	54
1110	53
1111	238

**TABLE 4E:** Autonomous motivation, with *N* = 482, *k* = 10 items, and *u* = 59 unique response patterns (Appendix B).

Pattern	Frequency	Pattern	Frequency
0000000000	89	0101011100	1
0000000001	5	0101111101	1
0000000010	1	0110011110	1
0000000100	1	0111000001	1
0000000110	1	0111111111	1
0000001100	1	1000000000	1
0000001111	1	1000000001	1
0000010000	5	1001111111	1
0000010001	2	1011111111	1
0000010011	1	1100000010	1
0000011000	7	1100111110	1
0000011001	3	1101011110	2
0000011010	1	1101111110	4
0000011011	1	1101111111	5
0000011100	21	1110011111	3
0000011101	7	1111000011	1
0000011110	12	1111100000	5
0000011111	4	1111100001	6
0000100000	1	1111100010	3
0001010000	1	1111100011	2
0001010010	1	1111101110	4
0010100011	1	1111101111	4
0100000000	5	1111110010	1
0100001110	1	1111110111	1
0100010000	1	1111111000	1
0100011000	1	1111111010	1
0100011110	6	1111111101	1
0100011111	4	1111111110	100
0100111110	3	1111111111	137
0100111111	2		

**TABLE 4F:** Controlled motivation, with *N* = 550, *k* = 9 items, and *u* = 134 unique response patterns (Appendix B).

Pattern	Frequency	Pattern	Frequency	Pattern	Frequency	Pattern	Frequency
000000000	114	010000110	6	100100111	1	110110100	1
000000001	5	010000111	2	100110011	1	110110110	2
000000010	16	010001110	1	101000000	1	110111110	1
000000011	2	010001111	1	101000001	3	110111111	4
000000100	33	010010010	1	101000011	1	111000000	2
000000101	3	010010100	1	101000111	3	111000001	2
000000110	28	010100000	1	101001011	1	111000011	1
000000111	4	010100011	1	101011011	1	111000100	1
000001000	1	010100100	1	101100011	2	111000110	1
000001100	3	010100101	2	101100101	2	111000111	5
000001110	6	010100110	5	101100111	2	111001110	1
000001111	2	010101110	1	101101101	1	111001111	1
000010000	1	010111110	2	101110100	1	111010111	2
000010100	5	011000111	1	101111101	1	111011111	5
000010101	1	011100000	1	110000000	5	111100000	4
000010110	3	011111111	1	110000010	1	111100001	4
000010111	1	100000000	19	110000100	4	111100011	3
000011100	3	100000001	2	110000110	5	111100100	2
000011110	3	100000010	2	110000111	4	111100101	4
000011111	3	100000011	2	110001011	1	111100110	1
000100000	1	100000100	7	110001110	1	111100111	19
000100110	1	100000101	4	110001111	2	111101000	1
000101101	1	100000110	3	110010100	1	111101011	1
000111110	2	100000111	4	110010110	1	111101100	1
000111111	1	100001001	1	110011110	1	111101111	11
001000000	2	100001101	1	110011111	1	111110010	1
001000001	1	100001110	2	110100000	1	111110011	2
001000111	1	100010000	1	110100011	1	111110110	1
001010000	1	100010010	1	110100100	1	111110111	8
001100001	1	100010100	1	110100101	1	111111101	3
001111111	1	100010101	2	110100110	2	111111110	1
010000000	3	100011101	1	110100111	2	111111111	49
010000010	4	100011110	1	110101110	4		
010000100	4	100100000	1	110101111	1		

**TABLE 4G:** All regulations, with *N* = 180, *k* = 19 items, and *u* = 109 unique response patterns (Appendix B).

Pattern	Frequency	Pattern	Frequency	Pattern	Frequency
0000000000000000000	15	0000011111100111111	1	1111110000111100000	1
0000000000000000010	3	0001000001000001110	1	1111110001111000001	1
0000000000000000100	3	0100000000111000111	1	1111110001111000100	2
0000000000000000110	7	0100000101111000111	1	1111110001111100101	2
0000000000000000111	1	0100001000000000000	1	1111110010000100111	1
0000000000000001100	2	0100001011111111110	1	1111110011111000000	1
0000000000000001110	1	0100011000000000010	1	1111110101111101011	1
0000000000000011100	1	0100100001111000100	2	1111110111111000011	1
0000000000000011110	1	0100100001111110111	1	1111110111111000111	1
0000000000000100001	1	0100100011111011110	1	1111110111111100011	1
0000000000000100111	1	0100111001111111111	1	1111110111111100101	1
0000000000001000000	1	1000000000000000000	1	1111110111111100111	1
0000000000010000000	1	1000000010000101101	1	1111111000001000000	1
0000000000110011111	1	1101000001111001111	1	1111111001111000000	1
0000000001000000010	1	1101101011111000100	1	1111111001111000100	1
0000000001101000100	1	1101111011111111111	1	1111111001111100100	1
0000000001110000000	2	1101111101111000001	2	1111111011111000011	1
0000000001110010100	1	1101111101111110111	1	1111111011111000101	1
0000000001111010110	2	1110011111111111111	1	1111111100111110111	1
0000000001111111111	1	1111100000000000101	1	1111111101111100000	1
0000001001000000110	1	1111100000001000000	1	1111111101111111111	1
0000001001111000100	1	1111100001111000000	6	1111111110001101111	1
0000001011110000110	2	1111100001111000001	1	1111111111111000000	1
0000010000000001001	1	1111100001111000010	1	1111111111111000111	3
0000010000000001110	1	1111100001111000011	1	1111111111111010010	1
0000010001000000111	1	1111100001111000100	3	1111111111111010111	1
0000010001100110101	1	1111100001111000101	1	1111111111111100001	2
0000010001110001101	1	1111100001111010000	1	1111111111111100100	2
0000010001110011110	1	1111100001111010100	1	1111111111111100111	1
0000010001110100100	1	1111100001111100001	1	1111111111111101000	1
0000010001110100111	1	1111100101111000001	1	1111111111111101111	2
0000010001111001110	1	1111101000111000111	1	1111111111111110011	1
0000010111111000101	1	1111101001111000010	2	1111111111111110111	3
0000011001110010100	1	1111101001111001110	1	1111111111111111101	2
0000011011110010100	1	1111101101111100111	1	1111111111111111111	24
0000011101111111111	1	1111101111111111111	1		
0000011111000111111	1	1111110000111000110	1		

**TABLE 4H:** For *A* = {*i*_1_, *i*_3_, …, *i*_5_, *d*_5_, *j*_1_, …, *j*_4_, *e*_3_, *e*_4_, *e*_5_}, with *N* = 342, *k* = 12 items, and *u* = 130 unique response patterns (Appendix B).

Pattern	Frequency	Pattern	Frequency	Pattern	Frequency	Pattern	Frequency
000000000000	32	000011000011	1	101101010100	1	111110001000	2
000000000001	1	000011000100	1	101111011111	1	111110001101	2
000000000010	8	000011000110	2	101111100001	2	111110010000	1
000000000100	24	000011010100	1	101111101111	1	111110011111	1
000000000110	29	000011011110	1	101111110000	1	111110101011	2
000000000111	3	000011100110	1	110011011111	1	111110110011	1
000000001001	1	000011100111	1	110011111111	1	111110110111	1
000000001111	2	000011101110	1	111000000000	1	111110111011	1
000000010111	1	000011101111	2	111011010110	1	111110111101	1
000000100000	1	000011110110	1	111100000000	19	111110111111	1
000000100111	1	000011111110	1	111100000001	1	111111000000	3
000001000000	1	000011111111	3	111100000010	1	111111000100	1
000001000100	3	000100000100	2	111100000011	1	111111001100	1
000001000110	3	000100001111	1	111100000100	5	111111001101	1
000001000111	2	000100010110	1	111100000101	3	111111010011	1
000001001001	1	000111000110	1	111100000110	2	111111010101	1
000001010110	4	000111001111	1	111100000111	1	111111010110	1
000001011110	1	001000000110	1	111100001001	1	111111011110	1
000001110000	1	001010000101	1	111100010000	1	111111011111	1
000001110010	1	001010001101	1	111100100000	2	111111101000	1
000010000001	1	001111001101	1	111100100001	1	111111101111	10
000010000100	1	011111111011	1	111100111101	1	111111110000	2
000010000101	2	100000000000	1	111101000000	1	111111110010	1
000010000110	3	100000000100	1	111101000010	2	111111110111	7
000010000111	1	100000000110	1	111101000110	1	111111111000	2
000010001100	2	100000010110	1	111101000111	1	111111111001	2
000010001101	1	100000011101	1	111101010000	1	111111111011	2
000010001110	2	100010000010	1	111101101111	1	111111111100	2
000010001111	2	100010111101	1	111101111111	1	111111111101	4
000010010000	1	100011110011	1	111110000000	7	111111111110	1
000010101111	1	101000000111	1	111110000001	1	111111111111	39
000010110101	1	101011111111	1	111110000100	2		
000011000010	1	101100000000	1	111110000110	1		

**TABLE 4I:** For *B* = {*i*_1_, …, *i*_5_, *d*_1_, …, *d*_4_}, with *N* = 577, *k* = 9 items, and *u* = 52 unique response patterns (Appendix B).

Pattern	Frequency	Pattern	Frequency
000000000	105	011001111	1
000000001	1	011100000	1
000000010	1	011111111	2
000000011	1	100000000	2
000000100	1	100111111	1
000000110	1	101111111	1
000000111	1	110000000	1
000001000	10	110000001	1
000001001	1	110001111	1
000001100	12	110010011	1
000001101	2	110011111	1
000001110	31	110101111	2
000001111	22	110111111	10
000010000	1	111001111	4
000101000	1	111100001	1
000101001	1	111101111	1
001010001	1	111110000	11
010000000	5	111110001	8
010000111	1	111110111	11
010001000	1	111111001	2
010001100	1	111111010	1
010001110	1	111111011	1
010001111	11	111111100	1
010011111	5	111111101	1
010101110	1	111111110	1
010111110	1	111111111	289

**TABLE 4J:** For *C* = {*d*_5_, *j*_1_, …, *j*_4_, *e*_1_, *e*_2_, *e*_5_}, with *N* = 706, *k* = 8 items, and *u* = 114 unique response patterns (Appendix B).

Pattern	Frequency	Pattern	Frequency	Pattern	Frequency	Pattern	Frequency
00000000	273	01010010	1	10100100	1	11011011	1
00000001	19	01010110	1	10101001	4	11011100	1
00000010	11	01011001	1	10101011	1	11011110	2
00000011	2	01011011	1	10101111	1	11011111	2
00000100	14	01011110	1	10110001	3	11100000	2
00000101	1	01101001	2	10110100	1	11100001	4
00000110	7	01110000	1	10111001	5	11100111	1
00000111	2	01110001	1	10111011	1	11101000	2
00001000	2	01111111	1	10111111	1	11101001	8
00001001	3	10000000	29	11000000	14	11101010	1
00001101	1	10000001	10	11000001	1	11101011	1
00001110	1	10000010	3	11000010	1	11101101	2
00001111	1	10000011	2	11000011	3	11101111	4
00010000	4	10000100	3	11000100	2	11110000	3
00010001	1	10000101	1	11000101	1	11110001	16
00010110	1	10000110	1	11000110	1	11110011	1
00010111	1	10000111	1	11001000	4	11110100	2
00011011	1	10001000	8	11001001	1	11110101	1
00011110	1	10001001	4	11001011	1	11110111	1
00100000	2	10001100	1	11001100	1	11111000	5
00100001	2	10001101	1	11001110	1	11111001	25
00100100	1	10010000	2	11001111	1	11111010	2
00110001	1	10010001	1	11010000	3	11111011	12
00111111	1	10010110	1	11010001	4	11111100	1
01000000	20	10011001	1	11010010	1	11111101	11
01000001	2	10011011	1	11010100	1	11111110	1
01000010	1	10100000	1	11010111	2	11111111	50
01000100	2	10100001	4	11011000	3		
01010000	15	10100010	1	11011001	1		

In general, IITA is supposed to provide more reliable results with larger sample sizes. In practice, however, a solution computed for a smaller sample size can still be an empirically useful model. This may be the case if the derived IITA hierarchy yields an acceptable and also simple description of a higher number of motivation variables, scaled altogether in one go. This may be especially so, if no other alternative model is available or difficult to get, or if an exploratory first model is needed that covers a larger number of variables to inspect for their multivariate relationships. Such a preliminary model may be further adjusted in subsequent analyses. A strategy for how to perform sequential exploratory IITA analyses with more and more refined subsets of motivation variables is outlined, actually for the first time in the literature on IITA, with the example of this section. Thus, we also contribute to the methodology of IITA. The results obtained are illuminating as they constructively add insight into a dispute in SDT literature (Chemolli and Gagné, 2014; Sheldon et al., 2017).

**FIGURE 4:**
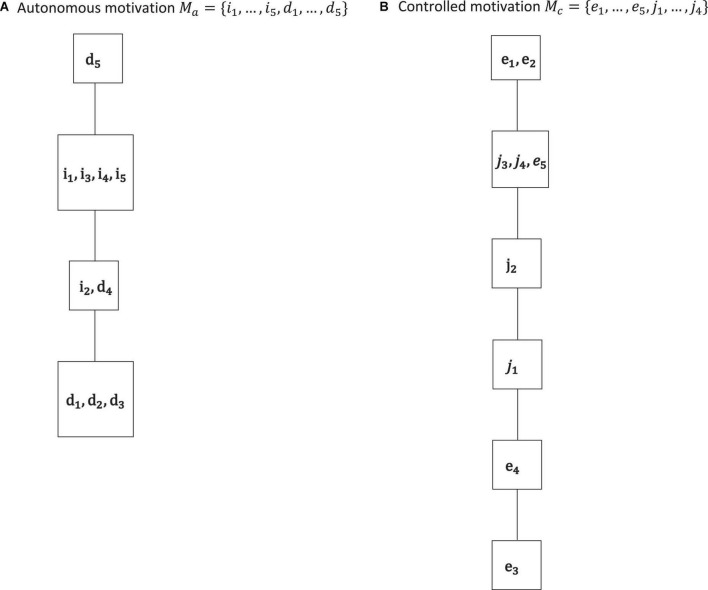
The IITA quasi-order solutions for autonomous motivation **(A)** and controlled motivation **(B)** of the empirical data set. By Birkhoff’s theorem, the corresponding quasi-ordinal motivation spaces are ℳ_*a*_ = {∅, {*d*_1_, …, *d*_3_}, {*i*_2_, *d*_1_, …, *d*_4_}, {*i*_1_, …, *i*_5_, *d*_1_, …, *d*_4_}, *M*_*a*_}, and ℳ_*c*_ = {∅, {*e*_3_}, {*e*_3_, *e*_4_}, {*e*_3_, *e*_4_, *j*_1_}, {*e*_3_, *e*_4_, *j*_1_, *j*_2_}, {*e*_3_, …, *e*_5_, *j*_1_, …, *j*_4_}, *M*_*c*_}, respectively. We see cumulative gradations of autonomous motivation and controlled motivation. This corroborates the higher-order interpretation of the basic motivations in these two non-basic autonomous and controlled motivations, which is also advocated in SDT literature.

**FIGURE 5:**
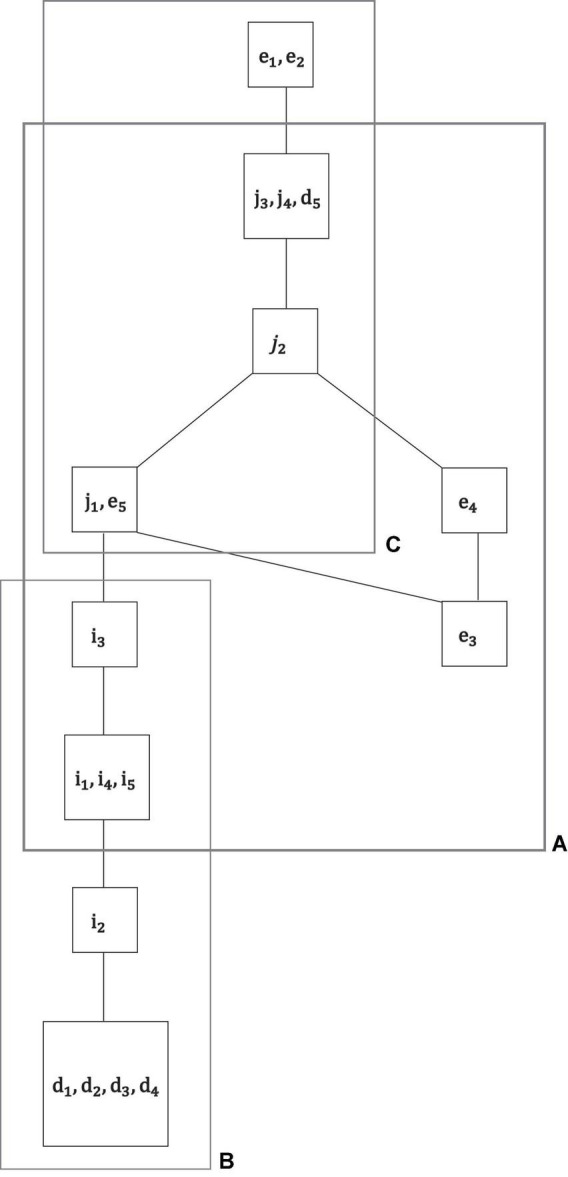
For exploratory analysis, all motivations of the empirical data set were jointly analyzed *M* = *M*_*i*_ ∪ *M*_*e*_ ∪ *M*_*d*_ ∪ *M*_*j*_. The sample size of *N* = 180 students for a total of *k* = 19 motivations was small for an overall IITA computation. Thus, based on the exploratory global solution (this figure), collections of motivations were delineated that were further analyzed more reliably, in lower numbers of jointly scaled motivations with larger sample sizes. In this example, the three subsets of motivations *A*, *B*, and *C* were regarded to be adequately sized and located within the overall graph for subsequent, more refined IITA analyses (Figures 6A–C).

**FIGURE 6:**
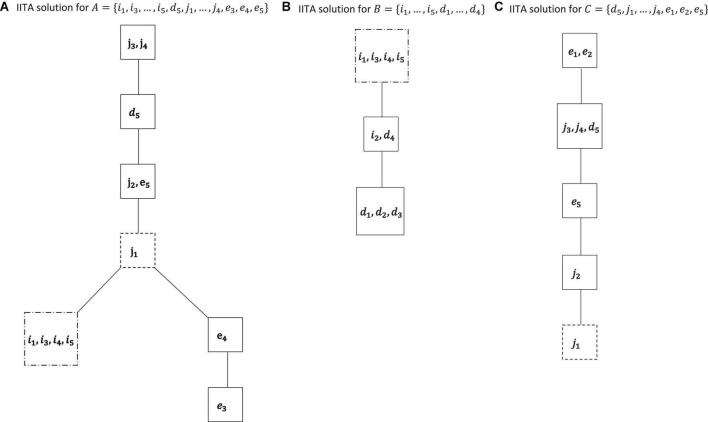
IITA computations for the motivations *A* = {*i*_1_, *i*_3_, …, *i*_5_, *d*_5_, *j*_1_, …, *j*_4_, *e*_3_, *e*_4_, *e*_5_} **(A)**, *B* = {*i*_1_, …, *i*_5_, *d*_1_, …, *d*_4_} **(B)**, and *C* = {*d*_5_, *j*_1_, …, *j*_4_, *e*_1_, *e*_2_, *e*_5_} **(C)** of the empirical data set. The dashed boxes indicate the respective linkage knots for the three graphs (Figure 7).

A remark regarding notation is in order. The manifest items or indicators of the instruments and their measured or underlying motivations are denoted with the same symbols. For example, we use *e* to stand for a manifest test item of the external regulation subscale and the corresponding latent external regulation. In particular, the variable names of the IITA solutions, for example, presented in the plots, are understood to be denoting the underlying motivations, not indicator variables. In the sequel, the five indicator variables and their corresponding (possibly equal) gradations of intrinsic regulation are *i*_1_, …, *i*_5_ (*o* = 5); for external regulation the manifest and latent variable names are *e*_1_, …, *e*_5_ (*l* = 5); and for identified regulation *d*_1_, …, *d*_5_ (*n* = 5) and introjected regulation *j*_1_, …, *j*_4_ (*m* = 4). Thus, the motivation domains for the poles of the internalization continuum are *M*_*i*_ = {*i*_1_, *i*_2_, *i*_3_, *i*_4_, *i*_5_} and *M*_*e*_ = {*e*_1_, *e*_2_, *e*_3_, *e*_4_, *e*_5_}, and for the intermediate regulations *M*_*d*_ = {*d*_1_, *d*_2_, *d*_3_, *d*_4_, *d*_5_} and *M*_*j*_ = {*j*_1_, *j*_2_, *j*_3_, *j*_4_}. We performed IITA analyses and derived sets and relations for the individual subscales (Figure 3). We also distinguished and ran the analyses separately in autonomous and controlled motivations *M*_*a*_ = *M*_*i*_ ∪ *M*_*d*_ and *M*_*c*_ = *M*_*e*_ ∪ *M*_*j*_, respectively (Figure 4). In addition, all motivations were jointly analyzed *M* = *M*_*i*_ ∪ *M*_*e*_ ∪ *M*_*d*_ ∪ *M*_*j*_ (Figure 5). Motivation models for further subsets of the domain were also derived (Figure 6).

**FIGURE 7:**
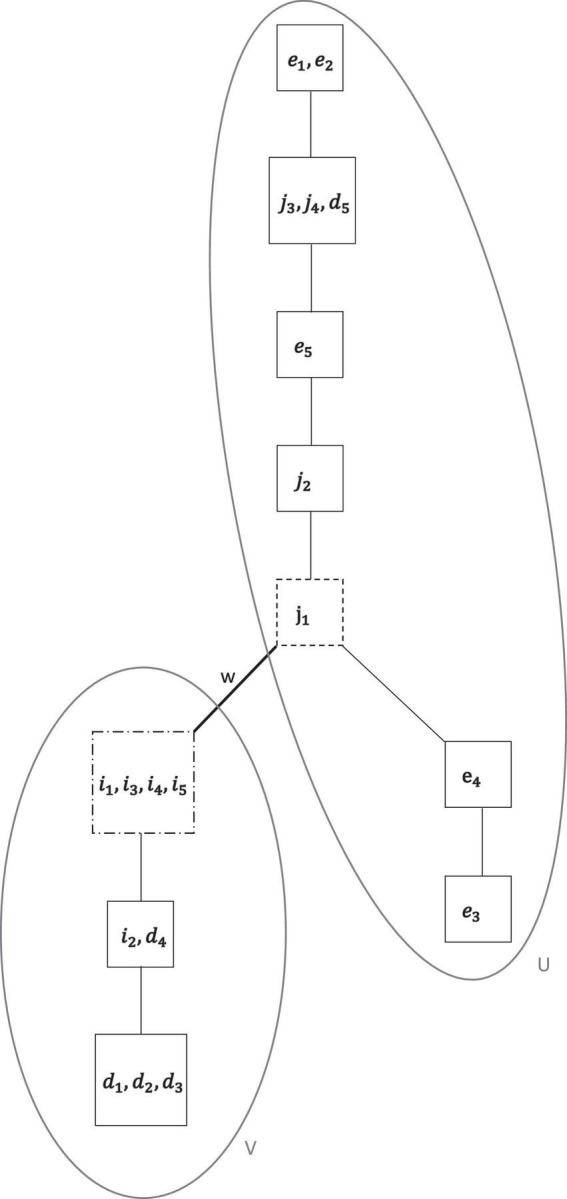
The concatenated final motivation co-occurrence relation ⊑_*f*_ on the motivation domain of all regulations *M* = {*i*_1_, …, *i*_5_, *d*_1_, …, *d*_5_, *j*_1_, …, *j*_4_, *e*_1_, …, *e*_5_}, obtained by sequential exploratory IITA analyses of the data set. The graph is composed of two, connected by *w*, chains *U* (of essentially controlled motivation) and *V* (of autonomous motivation).

Those motivations are interpreted as the gradations of the types of intrinsic regulation, external regulation, identified regulation, and introjected regulation, or of autonomous motivation and controlled motivation, which may or may not be a same gradation, depending on the solution obtained by IITA, in the following sense. Whether two motivations denominate the same gradation will be judged on the basis of parallel or equally informative motivations. Given the data-analytically derived motivation co-occurrence relation by IITA on *M* or *M*_*x*_, where *x* = *i*, *e*, *d*, *j*, *a*, *c*, we can obtain the corresponding quasi-ordinal motivation space. Any (two or more) regulations in *M* or *M*_*x*_, which are then contained in the same motivation states of this space, are called parallel or equally informative. In a sense, they carry the same information regarding the distribution of motivation in the population. Equivalently, in the other representation, parallel or equally informative regulations imply each other in the motivation co-occurrence relation. That is, for any two equally informative gradations, each one is a possessed motivation if and only if the other is. Equally informative gradations occur always jointly. We take this to mathematically define those parallel gradations or regulations of the solution that are “equal.”

A remark on this definition of “equality” is in order. Obviously, if two regulations as true constructs are equal objects, they necessarily, by identity, must be parallel. However, the converse may not be the case. One could imagine equally informative gradations being different constructs. But this is only hypothetical. In reality, by definition, parallel gradations can only be observed jointly. They cannot be separated empirically if the model holds true. In this sense, experimentally, we can only know their summative effect on another substantive variable of a study, but we have no information about what effect an individual part or gradation may have. This is reminiscent of entanglement in quantum mechanics in physics (e.g., Jaeger, 2009; Duarte, 2019), where we may know everything about a system, but nothing about its parts. If we cannot separate the parallel gradations, but only observe their “summative motivation,” which may be an aggregate “motivation” different from the postulated basic motivations, we can take any of those gradations to represent their same “summative motivation.” If this “summative motivation” is composed of only genuine gradations of the same basic regulation type, we assume or say that this “summative motivation” and its constituting parallel gradations are a same “gradation” of that regulation type.

To sum up, for practical purposes, if by data analysis, we detect two parallel regulations, letting aside variability of the solution, they may or may not be truly equal gradations (objects), which does not matter empirically, as a result of their unidentifiability, but in any case, they can be considered to be “equal” or equivalent in that more general sense. That is, if we term the latter notion of “equality” the parallel equality, in contrast to true (object) equality, the true equality cases entail parallel equality, but parallel equality encompasses additional cases that are not true equality. It is this generalized notion of equality that we use in the sequel.

In Figure 3, we can see the motivation co-occurrence relations, with their spaces below, that were detected for the separate subscales. We may expect a linear order since the items of each subscale should be measuring the same latent regulation type, in cumulative and possibly equal gradations.

The corresponding quasi-ordinal motivation spaces are, by application of Birkhoff’s theorem, ℳ_*i*_ = {∅, {*i*_2_}, {*i*_1_, *i*_2_}, {*i*_1_, *i*_2_, *i*_4_, *i*_5_}, *M*_*i*_}, ℳ_*e*_ = {∅, {*e*_3_}, {*e*_3_, *e*_4_}, {*e*_3_, *e*_4_, *e*_5_}, *M*_*e*_}, ℳ_*d*_ = {∅, {*d*_1_, *d*_2_, *d*_3_}, {*d*_1_, *d*_2_, *d*_3_, *d*_4_}, *M*_*d*_}, and ℳ_*j*_ = {∅, {*j*_1_}, {*j*_1_, *j*_2_}, {*j*_1_, *j*_2_, *j*_4_}, *M*_*j*_}. Except for introjected regulation, in each solution, we have equally informative regulations, namely, {*i*_4_, *i*_5_}, {*e*_1_, *e*_2_}, and {*d*_1_, *d*_2_, *d*_3_}. Thus, by definition (of parallel equality), we assume that these regulations denote equal gradations for each type of intrinsic regulation, external regulation, and identified regulation, respectively, and that the other gradations are distinct.

In SDT, researchers distinguish between autonomous (intrinsic and identified) motivations and controlled (external and introjected) motivations. This higher-order interpretation of the basic regulations is shown to be substantively important for the study of motivation and empirically adequate in motivation data (e.g., Deci and Ryan, 2008; Gagné et al., 2010, 2014). For example, evidence is reported by Gagné et al. (2010) that a second-order two-factor confirmatory factor analysis model can yield adequate fit, where the two higher-order factors group together the autonomous vs. controlled regulations as first-order factors. Consequently, in Figure 4, the relations and sets obtained by IITA in the autonomous and controlled motivations of the data set are reported.

Autonomous motivation and controlled motivation may be viewed as higher-order, aggregate types of motivation, different from SDT’s postulated basic motivations. The cumulative (i.e., linearly ordered) gradations of autonomous motivation and controlled motivation obtained in the solutions corroborate that interpretation. In particular, the results indicate that the intermediate regulations are interweaved with their respective polar regulations in their common aggregate types, fairly arbitrary. The former can imply the latter and vice versa. They can be equally informative gradations of their underlying higher-order regulation types, even if they are different basic motivations. For example, intrinsic motivation *i*_*2*_ and identified motivation *d*_*4*_, basic motivations of different subscales, are equally informative, and by definition, they may be viewed as the same gradation of autonomous motivation. Or, the parallel regulations *j*_*3*_, *j*_*4*_, and *e*_*5*_, if the model is true, can be viewed to be an equal gradation of the controlled motivation type. It is important to note, however, that the interpretation of the relations and their derivation from data are based on motivation possession or occurrence. Thus, possession of intrinsic motivation *i*_*2*_ implies the possession of one, and thus all, of the identified motivations *d*_*1*_, *d*_*2*_, and *d*_*3*_. External regulation *e*_*1*_ occurs if and only if external regulation *e*_*2*_ occurs, and in this case, all other regulations, external and introjected, must necessarily be also possessed motivations. Thus, if a person attains specific polar or intermediate regulations, we may infer that this person also possesses the motivations implied by those regulations.

In Figure 5, we ran the IITA algorithm on all motivations available in the data set for exploration, first and foremost. The sample size of *N* = 180 is small, relative to a large number of *k* = 19 motivation variables. That is okay since the aim was to look for possible interrelationships among the variables and to gain guidance on what motivation combinations to further investigate in narrow analyses.

In this example, we carved out the three subsets of motivations *A*, *B*, and *C* in Figure 5, which basically give a covering of the whole graph with overlapping motivations that will be used to mesh together the separate IITA solutions in those subsets. The choice of subsets made in Figure 5 was also motivated by the following observations. Motivation *e*_*5*_ seemed to be critical in the overall solution compared with what we obtained in Figures 3B, 4B. Also, motivations *d*_*5*_ and *i*_1_, *i*_3_, *i*_4_, *i*_5_ constituted linkages of autonomous motivation with controlled motivation, which is important information not contained in Figures 3, 4. In addition, subsets *B* and *C* are chains, indicating that each of their motivations is more strongly interrelated, where *B* is a proper subset of the autonomous motivation scale and deviates from the relation obtained in Figure 4A. The binding subset between *B* and *C* is *A*, where the external motivations are not in accordance with the solution computed in controlled motivation in Figure 4B. Thus, these subsets with their extra or deviating structures may require further consideration.

The refined analyses in those subsets *A*, *B*, and *C* are reported in Figure 6.

The dashed boxes of two types indicate the knots where the three graphs are lumped together, respectively. For solutions *A* and *B* of Figure 6, there is only one option, to append solution *B* to solution *A*. For solutions *A* and *C* of Figure 6, the more refined solution *C* replaces the corresponding part of solution *A*. This also turned out to be the more preferential meshed solution in accompanying data analyses in even smaller subsets of the involved variables. Thus, the combined and final motivation co-occurrence relation in Figure 7 was obtained.

**FIGURE 8:**
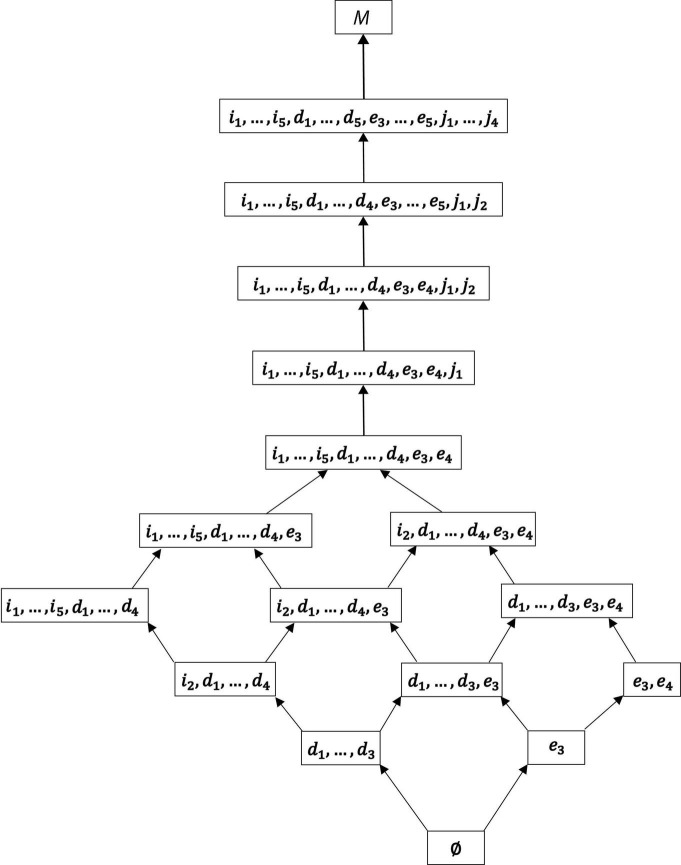
The quasi-ordinal motivation space ℳ_*f*_ corresponding to the final IITA solution ⊑_*f*_. The graphical representation is read from bottom to top, where a sequence of arcs linking a motivation state to a state located top of it means that the former is a subset of the latter. The bottom part of the graph indicates a branched multidimensional structure, the top part a linear unidimensional (“mixed” dimensionality).

The corresponding space of motivation states for this overall solution, as a graph, is shown in Figure 8.

From Figures 7, 8, we can see how the different motivations are interrelated with each other in the interpretation of motivation possession. Deviations from a perfect chain structure are due to incomparability between (all) intrinsic regulations (*i*_1_, …, *i*_5_) and (four) identified regulations (*d*_1_, *d*_2_, *d*_3_, *d*_4_) on the one hand and (two) external regulations (*e*_3_, *e*_4_) on the other, located in the bottom parts of these figures. Except for (one) identified motivation (*d*_*5*_), which is intermingled with (two) introjected motivation (*j*_3_, *j*_4_), the hierarchy depicted in Figure 7 can essentially be partitioned into the two chains of autonomous motivation (*V*) and controlled motivation (*U*). These parts are connected by an edge (*w*) between intrinsic motivation (*i*_1_, *i*_3_, *i*_4_, *i*_5_) and introjected motivation (*j*_*1*_).

We have the following interpretation, in our example, regarding the discrimination or separability of controlled motivation and autonomous motivation. According to the hierarchical structure of their defining regulations, in Figure 8, controlled motivation and autonomous motivation cannot always be mutually exclusive, and thus separable. There are the motivation states, which only entail either controlled motivation or autonomous motivation, {*e*_3_} and {*e*_3_, *e*_4_} or {*d*_1_, *d*_2_, *d*_3_}, {*i*_2_, *d*_1_, *d*_2_, *d*_3_, *d*_4_}, and {*i*_1_, *i*_2_, *i*_3_, *i*_4_, *i*_5_, *d*_1_, *d*_2_, *d*_3_, *d*_4_}, respectively. However, the majority of the states imply autonomous as well as controlled motivations jointly. For example, the state {*i*_2_, *d*_1_, *d*_2_, *d*_3_, *d*_4_, *e*_3_} is autonomous motivations *i*_*2*_ and *d*_1_, *d*_2_, *d*_3_, and *d*_*4*_ as well as controlled motivation *e*_*3*_. Thus, in a population of reference, depending on the distribution of the motivation states, we may end up sampling students either autonomously or controlled motivated (three or two states, respectively), autonomously as well as controlled motivated (eleven states), or none of them (∅).

Importantly, the final solution can be utilized for the qualitative assessment of combinatorial dimensionality, in the following sense. We see that the derived multidimensional structure, that is, genuine quasi-order, is close to a unidimensional structure, meaning a chain. In particular, note that this is not the common definition of numerical dimensionality of the parameter or factor space of a statistical (e.g., item response or structural equation) model. Compared to the latter, the combinatorial view of dimensionality is more qualitative. In the solution, in Figure 7, omitting the external gradations *e*_*3*_ and *e*_*4*_, on *M*\{*e*_3_, *e*_4_}, the restricted motivation co-occurrence relation is a single chain. Thus, the two-dimensional structure (i.e., linked two chains) of autonomous motivation *V* and controlled motivation *U* on the entire motivation domain becomes a unidimensional structure, if slightly pruned. That is, with the approach of this article, based on relations, we may see how close a multidimensional structure is to unidimensionality. In the empirical example it is very close, and where discrepancies in the structure may occur combinatorially. This may especially be so in relatively structured motivation data of validated SDT questionnaires.

In particular, this could dissolve a dispute of opinions put forth by Chemolli and Gagné (2014) and Sheldon et al. (2017). These authors advocated mutually exclusive views of a multidimensional vs. a unidimensional structure of motivation, respectively. In the empirical application of this article, at least, the two opinions only differ slightly, so both views basically seem to be justified in this example. In other applications, in the same manner, researchers could investigate the dimensionality of motivation. Presumably, if the theory holds true empirically, there should not be greater discrepancies between the unidimensional vs. multidimensional views of motivation, and this could be combinatorially quantified, similar to the example. However, this is only a conjecture, which needs to be tested critically in more popular scales; for example, in the Multidimensional Work Motivation Scale (MWMS), refer Gagné et al. (2010, 2014) and Trépanier et al. (2022).

To conclude, in Table 2, we summarize the frequency distributions of the dichotomous scores across motivation variables and the sample sizes underlying any of the IITA analyses of the empirical data sets. All analyzed data sets with individual binary entries can be found in Appendix B.

## 6. Usefulness of this approach for motivation research and limitation

We summarize the main conclusions from the results and the application of KST to SDT.

1. First and foremost, the problem of unidimensionality vs. multidimensionality of motivation, which has been disputed among SDT researchers (in particular, Chemolli and Gagné, 2014; Sheldon et al., 2017), can be more informatively assessed and resolved based on discrete KST combinatorial structures, as compared to numerical dimensionality of parametric psychometric models. Take as an example, Figure 7. It is by no means obvious, or unique, how to derive this figure’s hierarchy among the motivations by covariance structure models. You have too many fit and model selection indices (Tables 13.1 and 13.2 in West et al., 2012), and even if some of these indices may be indicative (but others generally are not) and you order motivations by their real unidimensional factor loadings, for instance, you may not be able to distinguish parallel or equally informative gradations nor account for the incomparable motivations (branching) located at the bottom part of the hierarchy. There may be workarounds though, more or less *ad hoc* and arbitrary solutions, which, however, may not be as straightforward and principled anymore as the natural combinatorial approach directly operating with orders.

2. In particular, we have contributed to the issue of the number of essential dimensions underlying the theory’s posited motivations. We could only try one data set. Thus, we conjecture that in other empirical data sets of validated SDT instruments, if occurring, multidimensionality, essentially, is two dimensions only, which are also qualitatively close to unidimensional. The findings of this article corroborated the higher-order classification into autonomous motivation and controlled motivation, which is also advocated by other SDT researchers, the two branches of the hierarchy, but altogether the motivations were also close to a single chain structure. Whether the close proximity of one vs. two combinatorial dimensions may entail significant differences in real use cases is an interesting question. If necessary, based on the hierarchy of dependencies among the regulations, combinatorially, we could exploit the full information in the graph and differentiate between “mixed” dimensionalities, in the sense that certain motivations are chained, thus combinatorially unidimensional, whereas others are branched, thus combinatorially two (or more) dimensional.

3. In addition, this article adds insight into possible inconsistencies that may arise in applications of the theory in the way the different regulation types can be empirically distinguishable (e.g., integrated regulation seems to be difficult to identify). In particular, we conjecture that there may be equally informative or parallel gradations of the basic regulation types that may not be separable empirically, and thus remain unidentifiable or indistinguishable by experiments, most likely. Therefore, the generalized notion of equality, parallel equality, of the regulations of motivation can be adequate.

4. A key requirement for any statistical or mathematical model used to represent motivation qualitatively should be the flexibility that comes with representing structures. This is more the case with discrete structures than with numerical values. Numerical values such as factor loadings, factor scores, person abilities, or item difficulties, typical parameters of the structural equation or item response models, are more strongly aggregated numbers, implying their more restrictive natural linear orderings. The use of more general combinatorial structures such as surmise relations or even surmises functions (Doignon and Falmagne, 1999) allows for greater flexibility in the representing structures, for a more qualitative, thus diagnostic, conceptualization of motivation. Even a multidimensional numerical approach, which generally may be ambiguous and more difficult to interpret compared with a unidimensional model, can only yield partially ordered structures, by component-wise comparisons. In particular, partial orders do not differentiate parallel or equally informative motivations. Due to the use of more general KST structures, you can in principle describe more data sets in practice.

5. On top of that, in contrast to the commonly used psychometric approaches, the representing structures in KST are mathematically linked by theorems such as Birkhoff’s, offering additional flexibility in the choices of equivalent representations, depending on the targeted use cases. For example, in the Rasch model (Van der Linden and Hambleton, 1997; Chemolli and Gagné, 2014), ability and difficulty estimates provide essentially separate representations, at the levels of persons and items, respectively. There is no direct and interpretable connection between these real-valued point estimates. In contrast, the more differentiated set representation (motivation structure) used for the persons is mathematically connected, by Birkhoff’s theorem (Theorem 1), to the fine-grained order representation (co-occurrence relation) used for the motivations. The two levels, people and regulations, are combinatorially interrelated, which offers more ways for qualitatively representing and interpreting motivation-related results.

6. A cornerstone of KST is adaptive testing (for applications in education, see Falmagne et al., 2013). Originally, KST was developed and is predestinated for this purpose. Thus, the KST approach to SDT, as advocated by us, can provide the necessary framework to develop adaptive assessment procedures for motivation testing in SDT. To our knowledge, adaptive testing has not been addressed at all in the SDT literature. To motivate, consider Figure 7. Under this co-occurrence relation, for example, if we test that a student is intrinsically motivated, possessing motivation *i*_*2*_, we do not need to further test that student for the identified regulations *d*_1_, *d*_2_, *d*_3_, and *d*_*4*_, as possessing the latter is necessary, implied by possession of the former. Or, if a student is tested not to be externally motivated, not possessing regulation *e*_*5*_, we can infer that this student must also not possess the motivations *j*_3_, *j*_4_, *d*_5_, *e*_1_, and *e*_*2*_. Thus, the assessment can skip testing for those motivations. These are only simple illustrative examples. Dependency hierarchies accompanied by adaptivity of this sort can build the basis for efficient computerized adaptive assessment procedures for testing and also training motivation in various SDT application domains (e.g., to automate and efficiently test, or train, work motivation among employees of a bigger company).

7. Related to the preceding point, the KST approach to SDT also has the advantage that it can allow for dynamical motivation systems for the study of motivational behavior in time, particularly how motivation can progress in an orderly fashion, or perhaps become altogether unpredictable or even chaotic. The provision of a dynamical (including longitudinal) self-determination theory could be accomplished based on stochastic (learning) paths (Falmagne, 1993). The general idea underlying such an extension of SDT has been conceptually exemplified in section “Sets and relations among motivations,” with Figure 1.

8. Mathematical, not statistical, modeling is possible. Mathematical models of motivation, such as discrete combinatorial structures (not only of KST), may help to understand, from a purely theoretical viewpoint, the logical foundations of the theory. In particular, the order-theoretic and algebraic properties of self-determination may be derived and studied mathematically (Ünlü, 2022), thus providing principled definitions of the central concept of self-determination. This may improve on the theory since in SDT, self-determination is commonly “defined” in a more or less *ad hoc* manner, based on such descriptive scoring protocols as the relative autonomy index (Grolnick and Ryan, 1987), an index that was criticized (Chemolli and Gagné, 2014; Ünlü, 2016). Or, the important internalization continuum of SDT can be mathematically defined by orders (Figure 2B).

9. Sets and relations derived for different motivations, for example, in the domains of work, sport, or learning, could be combinatorially compared, at a qualitative level. This could aim at finding structural invariants of motivation across different application contexts of the theory of self-determination.

The KST approach is relatively universal. It is generally applicable in any context in which implications between, even abstract, information units are of interest. For example, information units can be the geometry questions of a mathematical literacy test, where we are interested in whether the mastery of a geometry question implies the mastery of other questions of the test. In our context, the information units are regulations, among which the implications in their interpretation of motivation possession are considered. In principle, the application presented in this article is generalizable to other (psychological) theories (with appropriate operationalizations), if the system of interest, its defining units of information, the implications among those units, and their interpretation are delineated as the objects of the study. Not-so-obvious applications of this approach to other fields include system failure analysis (e.g., of a nuclear plant), where the failure of a component of the system may imply the failure of other components. Or, in medical diagnosis, the system is the patient, the information units are represented by the symptoms, and a physician examines whether the presence of certain symptoms in the patient imply that of others.

The present article has an obvious limitation, in that only one scale and data set were analyzed, which can be found in Appendices A, B, respectively. In future studies, more popular scales such as the MWMS (Trépanier et al., 2022), Sport Motivation Scale (SMS; Pelletier et al., 2013), and Academic Motivation Scale (AMS; Vallerand et al., 1992) with corresponding data sets could be examined using the KST and IITA approaches. This is an important direction for subsequent work. On the one hand, it remains to be seen if in those application domains with their questionnaires and data sets similar results can be obtained. On the other hand, it would be interesting to see how domain-specific motivation spaces and co-occurrence relations obtained in different application contexts of SDT structurally compare with each other. This could give (qualitative) information about the properties, similarities, and differences, of work motivation, sport motivation, and academic motivation, for example. To accomplish such a program, at this point, we have a personal recommendation addressed to the field of SDT. In the future, SDT researchers could consider providing their most pertinent data sets in a publicly accessible database. That would greatly facilitate research of the sort reported in this article.

## 7. Conclusion

Chemolli and Gagné (2014) advocated the use of qualitative motivation in self-determination theory (SDT). The approach presented here to motivation, an application of knowledge space theory (KST), is inherently qualitative, based on sets and relations in motivation regulations. In particular, this methodology allows us to treat each regulation as a separate variable of the motivation domain (cf., Koestner et al., 1996), and to represent every person by her or his total motivational profile, the motivation state, of all regulations within the person (cf., Vansteenkiste et al., 2009). This approach is flexible and general. It incorporates combinatorial unidimensionality as well as multidimensionality of the possible motivation structures in a unified and natural manner by the use of linear orders (chains) and genuine quasi-orders, respectively. In the empirical application, we have seen that there are unidimensional substructures, such as that of autonomous motivation (Figure 4A) and controlled motivation (Figure 4B). We have also seen that the overall motivation structure was basically branched into autonomous motivation and controlled motivation and was two-dimensional in that sense (Figure 7). However, that motivation structure could be slightly pruned to become a chain, and thus unidimensional. Basically, two combinatorial dimensions reducible to one were observed.

In essence, these results are in accordance with, both, the seemingly contrary and exclusionary opinions expressed in the articles by Chemolli and Gagné (2014) and Sheldon et al. (2017), thereby bringing together and uniting their views. The conjecture is that in empirical data sets of reliable and valid SDT instruments if occurring, multidimensionality is essentially two dimensions that are qualitatively close to unidimensional. In future applications of this approach to other scales (e.g., Trépanier et al., 2022), one could check if this may be generally so. But in any case, one could study how close a multidimensional relational structure is to unidimensionality, and how to prune the structure accordingly, if possible.

The presented qualitative conceptualization of motivation based on KST can contribute to SDT in novel ways, practically and theoretically (cf. also section “Usefulness of this approach for motivation research and limitation”). Especially, adaptive assessment and training of motivation, ideally computerized, or the dynamical or longitudinal analysis of motivation progression in time, could be accomplished based on stochastic paths in empirically valid motivation spaces of the feasible motivation states (Figures 1, 8). This article paves the way for these and other fruitful and interdisciplinary contributions to the study of motivation from the viewpoint of knowledge axiomatization and assessment in education. We have only considered the basic, yet powerful, concepts and their interpretations in motivation. More work in this direction is needed, especially by applied SDT researchers, to relate the combinatorial structures of motivation to behaviors and experimental outcomes.

In the end, we would like to describe this contribution as a cross-disciplinary methodology, an application of knowledge modeling in education and mathematical psychology, KST, to the study of motivation in, amongst others, social and personality psychology, SDT. As such, the KST approach is, most probably, not familiar to applied researchers in SDT, but we think it is worth the effort. To our knowledge, the qualitative representation and the analysis of motivation based on discrete combinatorial structures, as proposed in this article, are interesting new views on the quantitative treatment of motivation in the theory of self-determination.

## Data availability statement

The original contributions presented in this study are included in the article/supplementary material, further inquiries can be directed to the corresponding author.

## Ethics statement

Ethical review and approval was not required for the study on human participants in accordance with the local legislation and institutional requirements. Written informed consent from the participants’ legal guardian/next of kin was not required to participate in this study in accordance with the national legislation and the institutional requirements. Written informed consent was not obtained from the individual(s), nor the minor(s)’ legal guardian/next of kin, for the publication of any potentially identifiable images or data included in this article.

## Author contributions

The author confirms being the sole contributor of this work and has approved it for publication.

## Appendix

### Appendix A. German modification Academic Self-Regulation Questionnaire (SRQ-A)

The SRQ-A questionnaire, in its original two versions, can be retrieved from https://selfdeterminationtheory.org/self-regulation-questionnaires/ (as of 30 October 2022). Validations of this scale were presented by Ryan and Connell (1989) and Deci et al. (1992). The adapted and supplemented version of the SRQ-A in German by Müller et al. (2007), which is used for the empirical application, can be retrieved from https://ius.aau.at/wp-content/uploads/2016/01/mui_fragebogen.pdf (as of 30 October 2022). We have decided not to translate this scale into English, for the following reason. The German modification seems to be more than a mere translation of the original SRQ-A. According to the authors, only nine of the seventeen items are reused, just translated, items of the original SRQ-A. Understandably, it has only been validated in the German language (Müller et al., 2007). Thus, to avoid the risk of any distortion of its validated properties by an unverified translation (e.g., from German to English), we reproduce this scale in its original form in Table 3. Other works could study possible translations of this German modification of the SRQ-A into different languages (such as English), to validate or invalidate it in further settings.

### Appendix B. Data sets

The binary data sets used for the empirical application are available in Tables 4A–4J, as frequency tables containing the observed response patterns with their respective absolute frequencies, for intrinsic regulation (Table 4A), external regulation (Table 4B), identified regulation (Table 4C), introjected regulation (Table 4D), autonomous motivation (Table 4E), controlled motivation (Table 4F), all regulations (Table 4G), and for the subsets of motivations *A* = {*i*_1_, *i*_3_, …, *i*_5_, *d*_5_, *j*_1_, …, *j*_4_, *e*_3_, *e*_4_, *e*_5_} (Table 4H), *B* = {*i*_1_, …, *i*_5_, *d*_1_, …, *d*_4_} (Table 4I), and *C* = {*d*_5_, *j*_1_, …, *j*_4_, *e*_1_, *e*_2_, *e*_5_} (Table 4J). Note that the sequence of how the response patterns are laid out in a data set according to their absolute frequencies is irrelevant for computations. The data sets made available in this appendix were analyzed by IITA with free software (Schrepp, 2006; Ünlü and Sargin, 2010).
